# Review Study on Mechanical Properties of Cellular Materials

**DOI:** 10.3390/ma17112682

**Published:** 2024-06-02

**Authors:** Safdar Iqbal, Marcin Kamiński

**Affiliations:** Department of Structural Mechanics, Faculty of Civil Engineering, Architecture and Environmental Engineering, Lodz University of Technology, 93-590 Lodz, Poland; safdar.iqbal@dokt.p.lodz.pl

**Keywords:** cellular solids, numerical modeling homogenization method, simulation processes, uncertainty quantification, finite element method, periodic solid media

## Abstract

Cellular materials are fundamental elements in civil engineering, known for their porous nature and lightweight composition. However, the complexity of its microstructure and the mechanisms that control its behavior presents ongoing challenges. This comprehensive review aims to confront these uncertainties head-on, delving into the multifaceted field of cellular materials. It highlights the key role played by numerical and mathematical analysis in revealing the mysterious elasticity of these structures. Furthermore, the review covers a range of topics, from the simulation of manufacturing processes to the complex relationships between microstructure and mechanical properties. This review provides a panoramic view of the field by traversing various numerical and mathematical analysis methods. Furthermore, it reveals cutting-edge theoretical frameworks that promise to redefine our understanding of cellular solids. By providing these contemporary insights, this study not only points the way for future research but also illuminates pathways to practical applications in civil and materials engineering.

## 1. Introduction

Cellular solids have attracted significant attention and made substantial advancements across various disciplines because of the variety of their porous periodic or irregular microstructure and lightweight properties. These materials possess desirable attributes such as high strength-to-weight ratios [1,2,3,4,5], high energy absorption capabilities [6,7,8], and thermal insulation properties [9], making them particularly valuable in civil and mechanical engineering. Their applications in this field range from structural components and insulation systems to lightweight filling materials [10,11,12,13,14,15].

Natural cellular materials provide innovative solutions for construction by replicating efficient and strong structures found in nature, such as bones and plants. These materials offer strength, lightweight properties, and energy-absorbing capabilities. By mimicking these natural designs, building materials with higher performance and sustainability can be developed, including applications such as lightweight panels, load-bearing structures, and insulation systems. The use of renewable and biodegradable materials is consistent with environmentally friendly building practices. Ongoing research in this area promises to transform buildings with high-performance and environmentally friendly alternatives [16,17,18].

The global economic impact of cellular solids in civil and materials has different quantitative analysis with normal material for the economic perspective, such as in terms of cost and saving in Mexico, the use of novel cellular concrete mixtures was found to reduce annual energy costs by 15% to 28% compared to conventional concrete [19], and the cellular concrete beams could lead to material cost savings ranging from 40% to 63% per beam compared to traditional box-shaped beams [20].

Cellular structures, both microscopic and macroscopic variants, are integral to numerous applications due to their unique properties. Microcellular structures are characterized by cell sizes in the nanometer to micron range and possess unique properties such as a high surface area to volume ratio and customized functionality due to their complex microscale structure. They have a wide range of uses in catalysis, filtration, and biomedical engineering, where precise control at the nanoscale is crucial. In contrast, macroscopic honeycomb structures, with larger cell sizes ranging from millimeters to centimeters, offer advantages such as lightweight structure, efficient energy absorption, and thermal insulation. They are widely used in the aerospace, automotive, and construction sectors and are used in applications such as sandwich panels, thermal insulation, and impact-absorbing structures. A comprehensive understanding of the design principles, fabrication methods, and performance characteristics of micro- and macro-cellular structures is critical to maximizing the potential of different disciplines, fostering innovation, and pushing technological boundaries [21,22,23,24].

In the fields of civil and materials engineering, the impact of cellular solid materials on the global economy has revealed quantitative analysis insights that distinguish them from traditional materials. Mexico is a notable case study where groundbreaking research has illuminated significant avenues for cost reduction. For example, investigations into innovative cellular concrete mixes have shown significant reductions in annual energy expenditures, ranging from 15% to 28%, compared to conventional concrete practices [19]. In addition, the use of cellular concrete beams has shown significant economic advantages, with potential material cost savings per beam ranging from 40% to 63% compared to conventional box beams [20]. These findings highlight the clear economic benefits of adopting cellular solid materials in civil engineering, not only in terms of improved energy efficiency but also in terms of significant reductions in material expenditures.

Mechanical properties of cellular materials, such as honeycombs, foams, or networks, including increased specific strength, stiffness, and high energy absorption capacity, make them suitable for applications involving impacts, collisions, or explosions [25,26,27,28,29,30,31,32,33]. Recently, network architectures within cellular structures have gained attention for their ability to outperform traditional designs in absorbing impulsive charges, allowing ample space for plastic deformations and efficient dissipation of impact forces. However, creating geometrically accurate cellular network structures remains a challenge and an open research problem. Some advances in additive manufacturing now enable the fabrication of intricate structures using customizable materials, offering exciting opportunities for design and optimization across various geometrical scales.

The significance of honeycomb structures in various fields, focusing on their structural capabilities, mechanical properties, and uses. Honeycomb structures are known for their hexagonal cell composition, which gives them an excellent strength-to-weight ratio, making them ideal for lightweight components in the aerospace, automotive, and construction industries. Their ability to efficiently absorb energy and high tensile strength make them suitable for applications requiring impact resistance. Ongoing research and development programs aim to enhance the mechanical properties, manufacturing processes, and environmental sustainability of honeycomb structures. Understanding the complex design principles and functions of these devices is critical to improving their efficiency and promoting technological advancement in different engineering fields [34,35,36,37,38].

Polymeric, metallic, and ceramic cellular materials have specific structural properties that make them ideal for a variety of technical applications. Polymeric cellular solids, such as polymethacrylimide (PMI) foams [39], are lightweight and have high energy absorption and thermal insulation capabilities, making them perfect for the automobile and aerospace sectors [40]. Metallic cellular structures [41], such as aluminum and titanium foams, provide high strength-to-weight ratios and excellent mechanical performance under load, which are essential for structural and impact-resistant applications. Ceramic cellular materials, such as silicon carbide foams, have excellent thermal stability, high-temperature resistance, and chemical inertness, making them ideal for use in thermal protection and filtration systems [3,42]. Advanced production processes, such as additive printing and high-precision molding, allow for fine control over the microstructure and porosity of these materials, improving their performance properties.

The engineers can tailor cellular materials to meet specific performance requirements, leading to aerospace, automotive, defense, and civil engineering innovations. Mathematical and numerical techniques facilitate the simulation and analysis of these materials, enabling rapid prototyping and optimization, and thus accelerating the development and deployment of advanced cellular solids solutions in real-world applications. Some research methods enabling the analysis of such media and their application areas [38,43,44,45] have been shown in Table 1.

Mechanical properties of cellular structures can also be discussed in the context of possible uncertainty in their geometrical parameters and/or mechanical properties of the original solid serving as the skeleton of the given unit cell [51,52,53]. Using various stochastic and probabilistic methods, scientists can predict and identify the behavior of cellular materials under various conditions in terms of basic probabilistic characteristics like expectations and variations, as well as the probabilistic entropy concept. Probabilistic models provide statistical information about the probability of various outcomes, including failure modes, fatigue life, and deformation characteristics, providing valuable information for reliability-based design optimization and also risk assessment [54,55,56]. Numerous studies have been undertaken to explore the mechanical properties of cellular materials through the application of stochastic and probabilistic analyses, and some of them are collected in Table 2.

It is seen that researchers are integrating mathematical and numerical models to clarify the underlying mechanisms that govern the behavior of cellular materials, thereby paving the way for advances in design optimization, predictive modeling, and material development. One of the most important observations from Table 1 and Table 2 is that, contrary to a number of Stochastic Finite Element Method (SFEM) studies with various solids and structures, now some non-Gaussian variables and processes become very important, so it needs more advanced computational algorithms.

Open-cell foam is characterized by interconnected pores that facilitate the free flow of fluids and gases within the material. Open-cell foam modeling involves the complex capture of the network of struts and ligaments that make up its structure, where geometric parameters such as pore size distribution and cell morphology can significantly affect its mechanical properties. Computational models play a crucial role in simulating various aspects of open-cell foam behavior, including deformation, compression, and failure mechanisms under different loading conditions. In contrast, closed-cell foams have isolated cells, requiring modeling approaches to account for their unique morphology and the presence of air bubbles within the cells. These closed-cell structures typically exhibit higher stiffness and strength compared to open-cell structures, which is attributed to the gas encapsulated within the pores. Computational models are used to analyze the elastic deformation, collapse behavior, and energy absorption capacity of closed-cell foam under different loading conditions. The difference between open-cell and closed-cell foam modeling in various research studies is demonstrated in Table 3.

Finite element analysis (FEA) has become an important tool for understanding and predicting the mechanical behavior of porous solids, providing crucial insights for engineering applications. This study provides a comprehensive overview of recent advances in applying FEA technology to various aspects of porous solids, including 3D structures with open pore configurations, polymethacrylimide (PMI) foam microstructures, and piezoelectrically active porous solids with different geometries. Furthermore, we discuss the integration of stochastic methods such as the stochastic finite element method (SFEM) for probabilistic analysis, coupling Voronoi structures with FEA models to study complex geometries, and analyzing programmable cellular solids. Furthermore, to discuss the implementation of the finite element method in software packages such as ABAQUS (version 2023, for instance) and its impact on modeling the mechanical behavior of cellular solids. Through these advancements, FEA continues to play a key role in optimizing the design and performance of porous materials and structures for diverse engineering applications.

The main aim of this review study is to elucidate the mechanical properties of cellular materials and solids within the framework of numerical and mathematical models. This work also explores the microstructure formation of cellular solids. In addition, it examines computer-based models including 3D Additive Manufacturing (AM) structures, Laguerre tessellation, 2D and 3D Voronoi diagrams, ABAQUS-based models, tetradecahedral (Kelvin) structures, in situ X-ray tomography Scanning, finite element modeling, and Bravais lattice systems to explain mechanical properties through homogenized equations. Furthermore, this review considers computational simulations and experimental results of cellular materials to gain a comprehensive understanding of their mechanical behavior.

This review work is subdivided into several sections, each addressing specific aspects. In Section 1, we provide an introductory overview of cellular materials, detailing their mechanical properties through various methodologies and also in this section describes the introduction of cellular material and the various tabulated forms of specification of cellular material, explained by various approaches to defining their mechanical properties. Section 2 discusses the widely applied microstructure of cellular solids through numerical and computational models. These include 3D AM (acoustic metamaterials) lattice structures with specific geometric patterns for controlling sound wave propagation, random tessellations of polycrystalline materials, Voronoi tessellations involving regular and perturbed hexagons, 3D Voronoi structures coupled with finite element (FE) models, tetrakaidecahedral (Kelvin) models of cellular solids based on FEA, polymethacrylimide (PMI) foam analysis via in situ X-ray computed tomography, Archimedean solids such as square and hexagonal folded octahedrons, and programmable materials featuring stretching-dominated and bending-dominated honeycomb unit cells. Section 3 We delve into the homogenization of mechanical properties within cellular solids, focusing on formulation aspects. Section 4 explores experimental investigations conducted on cellular solids, encompassing mechanical properties of programmable materials, buckling strength of 3D AM-based lattice unit cells, band frequency analyses, tessellation outcomes (gamma distribution), and the development of phenomenological and micro-mechanical models. Section 5 discusses computer simulations applied to cellular solids, including finite element analysis (FEA) of programmable cellular solids and 3D AM-based lattice structures, along with computing mean and standard deviation of surface areas and the analysis of transmission loops in kinematic cells. Meanwhile, Section 6 proposes mitigation strategies in the face of uncertainties associated with cellular materials. Section 7 consolidates discussions on numerical, mathematical, and computational simulations, and Section 8 concludes with an overview summary of findings and insights gained from numerical applications in this study of cellular solids.

## 2. Microstructure of Cellular Material

In industrial applications, tuning the stiffness of cellular materials by selectively removing material is a key strategy to achieve desired mechanical properties. Engineers utilize a variety of methods to achieve this, including advanced computational tools such as finite element analysis (FEA) combined with topology optimization algorithms. Through iterative simulations, material is strategically removed from the honeycomb structure to achieve an optimized stiffness distribution while ensuring mechanical integrity. In addition, gradient density structures are employed, where spatial variations in material density allow for local stiffness tuning. Additive manufacturing (AM) technologies play a key role, enabling the fabrication of complex honeycomb geometries with precise material distribution control. Auxetic structures exhibit unique negative Poisson’s ratio behavior, expanding laterally when stretched, providing enhanced stiffness and energy absorption properties. Engineers also utilize layered designs and hybrid material combinations to further fine-tune stiffness properties. Iterative design methods involving prototyping and testing ensure that honeycomb materials meet stringent mechanical requirements while optimizing weight and cost. Together, these strategies have led to the creation of lightweight, customized honeycomb materials to meet the specific needs of different industrial sectors, from automotive to aerospace to infrastructure.

Using contemporary techniques, the creation of cellular materials’ micro- and macrostructures can be thoroughly comprehended. The arrangement and connectivity of cells or pores define structures at the microscopic scale, and these structures are frequently modelled using sophisticated computational methods like digital image correlation (DIC) [75,76] and finite element analysis (FEA) [77,78,79,80]. By simulating and visualizing interior geometries with accuracy, these techniques shed light on how microstructural characteristics affect the overall properties of materials. Contemporary techniques additionally integrate additive manufacturing technology, such as three-dimensional printing [81,82], to fabricate intricate, regulated microstructures that emulate native biological materials. Homogenization procedures are used to close the gap between bulk material properties and microstructural behavior at the macroscale. This is predicting the mechanical reaction of the entire material by averaging its tiny qualities. By combining these multiscale methods and experimentally verifying them using imaging methods like computed tomography (CT) scanning [83] and mechanical testing, cellular materials can be fully understood and optimized for a range of engineering uses. These techniques help to clarify the connection between mechanical qualities and microstructure while also making it easier to design materials with unique characteristics in response to particular building requirements.

The literature devoted to the creation of cellular material microstructures is addressed in this section. Cellular materials are usually produced using two main techniques. One approach involves processing techniques, wherein objects with honeycomb or lattice patterns are designed using 3D printing and materials like polymers, metals, or ceramics. Foaming is a different processing method that creates cellular materials by adding gas or bubbles to a liquid or molten form before solidifying [45,84]. Some of the research work that defines types of cellular materials and the method of microstructure generation is shown in Table 4.

The second and most popular approach uses computational techniques to virtually create cellular materials, such as topology optimization and finite element analysis. Using these techniques, materials’ behavior under various conditions was simulated, and their microstructure was optimized to produce desirable characteristics like stiffness, strength, or thermal conductivity [46,90,91,92]. Some of the research studies that used computational techniques for the microstructure formation of cellular material are shown in Table 5.

**Figure 1:**
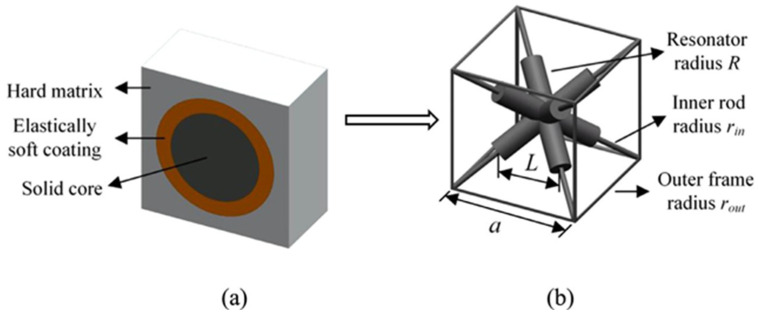
(**a**) Sectional view of the unit-cell of a 3D acoustic metamaterial, (**b**) design of the unit-cell of a 3D AM-based lattice structure [43].

**Figure 2:**
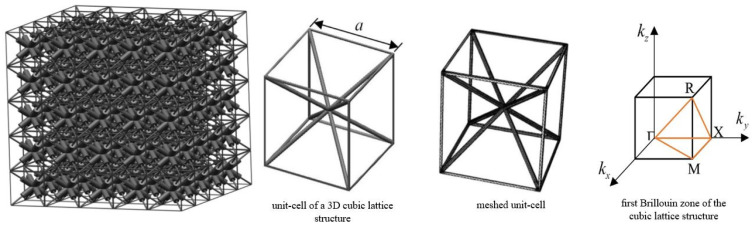
3D AM-based lattice structure [43].

The engineering materials known as “acoustic metamaterials” can stop elastic or acoustic waves from propagating within a given frequency range [43,100,101]. Localized elastic resonances are generally produced by a softer material encircling a dense core. This idea was modified to create a straightforward body-centered cubic lattice structure, as seen in Figure 1 and Figure 2. The lattice structures cause low-frequency resonances by creating a discontinuity in the unit cell’s cross-struts. The three primary components of this unit cell were the resonator, inner rods, and outer frame, as shown in Figure 2. This innovative approach allows us to fabricate lattice structures with low-frequency bandgaps, preventing elastic wave propagation within the material [43].

**Figure 3:**
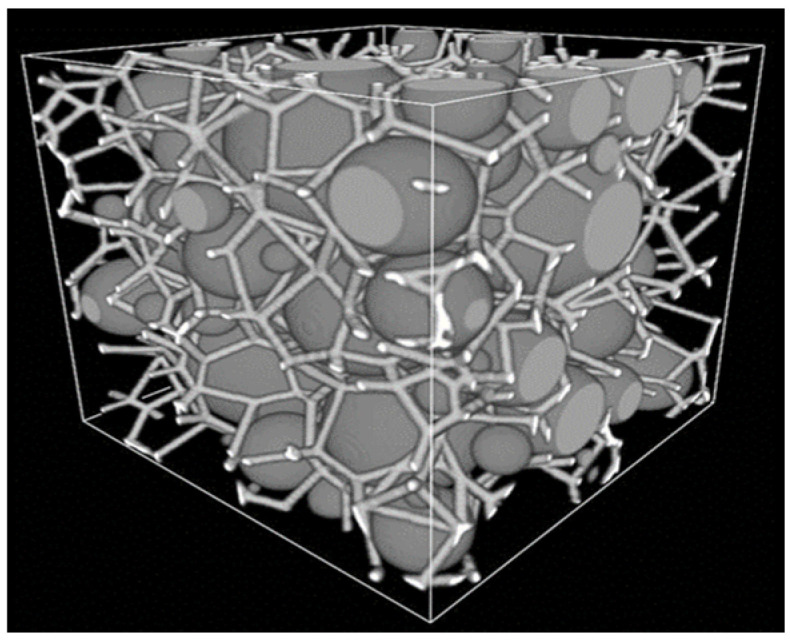
Visualizations of the edge system of a Laguerre tessellation of a dense packing of spheres together with some of the generating spheres [46].

As space-filling arrays of non-overlapping convex polytopes, random tessellations are frequently employed as models for polycrystalline or cellular materials [46]. The facets of these tessellations depict closed-cell foam, and the edges represent open-cell foam in foam structures. Unlike deterministic models like the Weaire–Phelan foams [102] and the Kelvin approach [103], they frequently fall short of adequately representing the variety of cell sizes and shapes found in solid foam structures. For modeling foam structures, non-overlapping spherical packing’s provide random Laguerre tessellations, which show great promise. The tessellations from sporadic sphere arrangements blend the regular and random features found in actual foams. Grain growth simulations for sintered materials demonstrate how polycrystalline materials can be modeled using Laguerre tessellations. The relevance of mosaics created by sphere fillings seen in Figure 3 [46] was further highlighted by the early stages of the sintering processes, which are characterized by models based on random sphere fillings.

**Figure 4:**
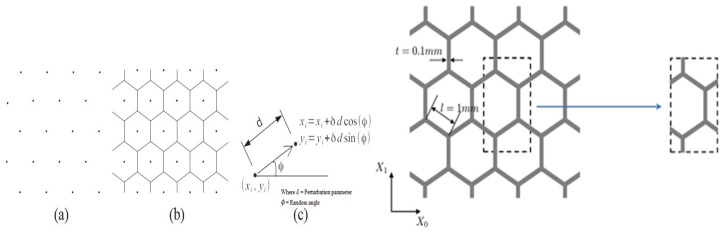
Voronoï diagram of the hexagonal honeycomb: (**a**) regular control points; (**b**) generated regular hexagons; and (**c**) coordinate perturbation at each control point i [35].

Some regular hexagonal honeycomb structures of the representative material cell may be used for numerical analysis—an example with wall length l = 1 mm and thickness t = 0.1 mm was analyzed by Nguyen & Noels (2014) [35] in this context. However, random generations are used frequently to create such a microstructure. As an example—the Voronoi tessellation technique is popular and was utilized to generate both regular and perturbed hexagons, as shown in Figure 4.

**Figure 5:**
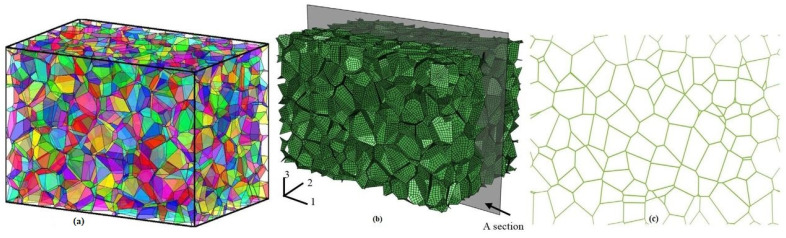
(**a**) 3D Voronoi structure; (**b**) Corresponding cell base on the FE model; and (**c**) middle section perpendicular to the 2nd direction [41].

Zheng et al. 2014 [41] used the Finite Element Method implementation in the system ABAQUS to study the cellular specimen having a volume of 30 × 20 × 20 mm with 600 nuclei, as shown in Figure 5.

**Figure 6:**
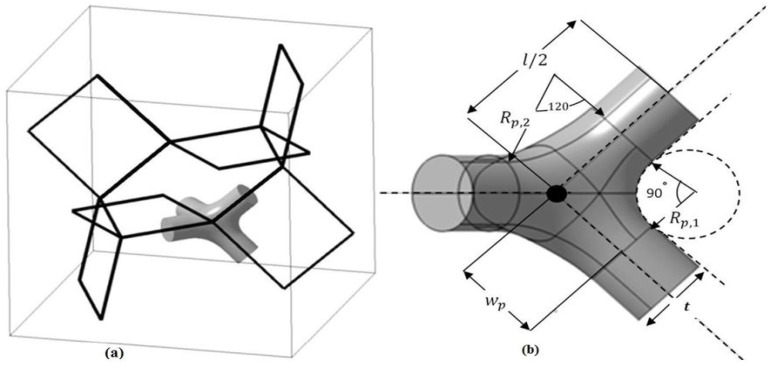
(**a**) Unit cell of tetrakaidecahedral (Kelvin) (**b**) geometry of a vertex [49].

**Figure 7:**
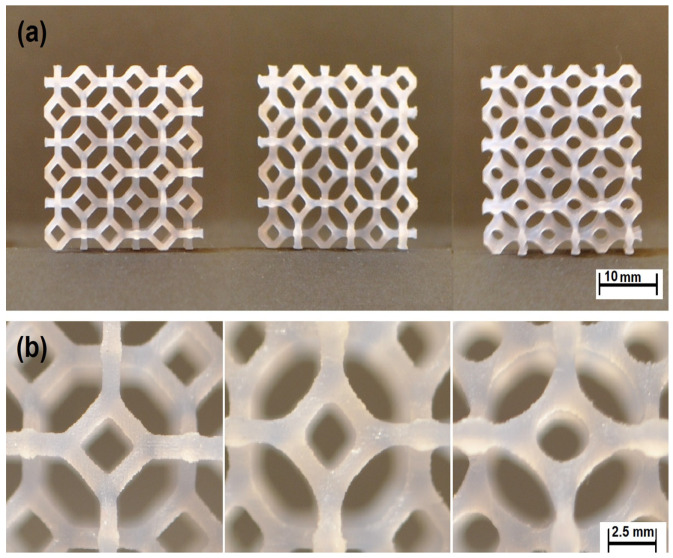
(**a**) Side view of the fabricated samples, (**b**) Magnified view of the distribution of solids in vertices [49].

In the work presented by Zargarian et al. [49], the elastic properties of cellular solids were discussed using the Finite Element Analysis (FEA) for the tetrakaidecahedral (Kelvin) unit cell. This unit cell comprises a 14-sided polyhedron composed of 6 square faces and 8 hexagonal faces, with 36 edges and 24 vertices. The joints in the unit cell are formed by two lines parallel to the center line of each edge, as represented Figure 6 and Figure 7.

**Figure 8:**
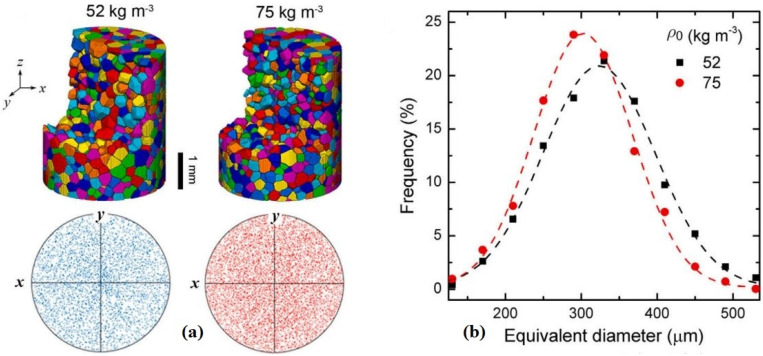
(**a**) CT characterizations of two foams with initial density *ρ*_0_ = 52 and 75 kg m^−3^ (**b**) Cell size (equivalent diameter) distribution. Symbols denote experimental data, and dashed lines fit with the Gaussian function [39].

Chai et al. 2020 [39], Polymethacrylimide (PMI) foams have high stiffness and strength compared to other foams. In situ X-ray computed tomography was used to examine the 3D microstructure properties under quasi-static compression. The computed tomography of cylindrical samples of two densities, 52 and 75 kg m^−3^, is shown in Figure 8.

**Figure 9:**
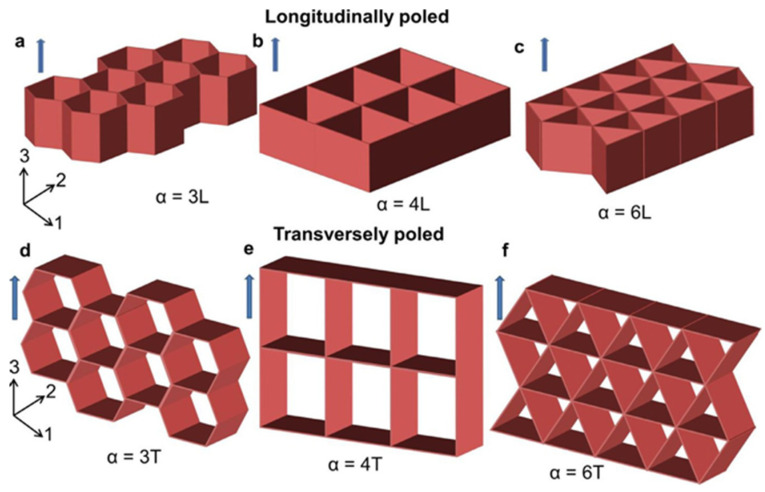
Schematics illustrate the piezoelectric cellular structures with nodal connectivity (α) of 3, 4, and 6, respectively (**d**–**f**), representing the honeycomb, tetragonal, and triangular structures studied in the present work. In the longitudinally poled structures (**a**–**c**) the porosity is aligned with the poling direction (i.e., three-direction), while in the transversely poled structures, the porosity is orthogonal to the poling direction [36].

Three basic cellular solid structures hexagonal, tetragonal, and triangular were studied by Iyer et al. [36] to illustrate piezoelectrically active cellular solids, highlighting their bending and stretching advantages. This study focuses on a two-dimensional honeycomb piezoelectric porous solid, as shown in Figure 9, by performing a comparative analysis using finite element modeling.

**Figure 10:**
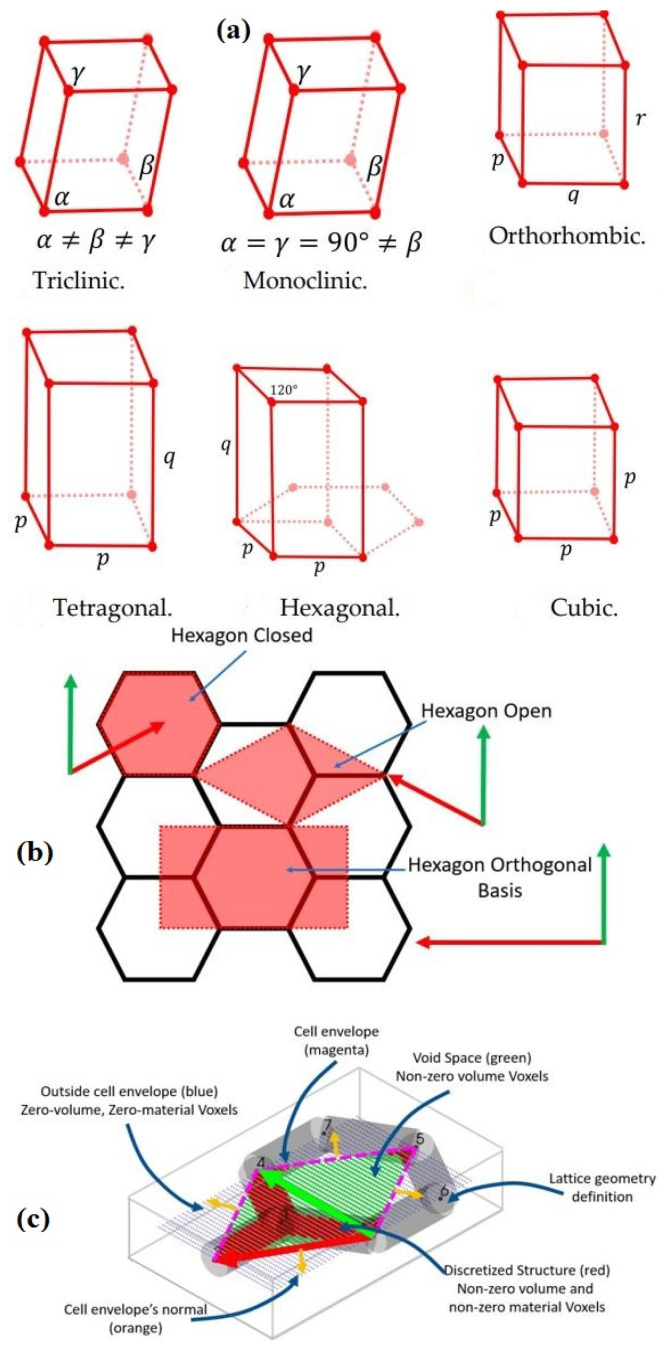
(**a**) Primitive Bravais lattices. *α*, *β*, and *γ* are angles (*α* ≠ *β* ≠ *γ*), whereas *p*, *q* and *r* are lengths (p ≠ q ≠ r). (**b**) Honeycomb lattice RVE with multiple cell envelope definitions. (**c**) Visualization of the 2D Open Hexagon RVE, RVE’s envelope, voxels, and periodic basis [37].

Rajakareyar et al., 2023 [37], discussed periodic cellular lattice structures classified based on the shape of the cell envelope. The envelope is an orthorhombic, tetragonal, or cubic shape as it belongs to the Bravais lattice system shown in Figure 10. Conversely, non-orthogonal envelopes with irregular shapes correspond with triclinic, monoclinic, or hexagonal systems. The representative volume element (RVE) of the lattice is the basic unit cell, consisting of the unit cell envelope and the lattice structure. It describes various representations of RVE, including orthogonal and non-orthogonal bases, using the honeycomb lattice shown in Figure 10. Furthermore, it presents a discrete 2D hexagonal geometry with non-orthogonal cell envelopes, highlighting three types of voxels. Among them, green voxels represent voids within the RVE envelope that contribute to volume calculations but are not included in calculations involving periodic node pairs and stiffness matrix determination.

**Figure 11:**
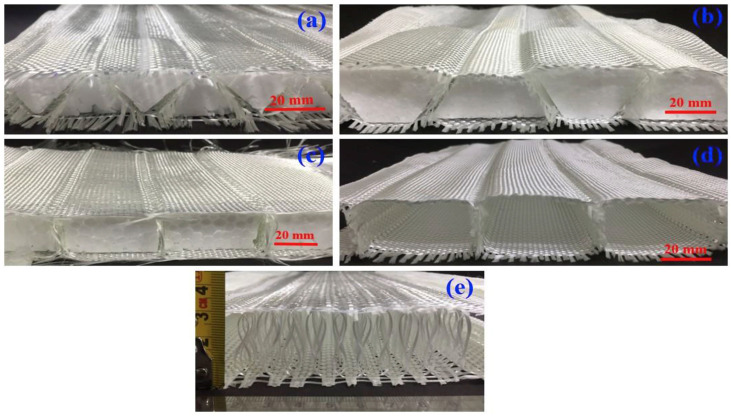
Fabric structures were produced for (**a**) TR, (**b**) TPZ, (**c**) RECTSL, (**d**) RECTDL, and (**e**) SPY spacer configurations [104].

The approach involves creating universal weave designs for various fabric structures such as triangular (TR), trapezoidal (TPZ), rectangular spacer RECTSL, rectangular spacer structure with double layer (RECTDL), and sandwich structure connected with core piles (SPY). These structures consist of two layers of skin fabric and a center fabric layer, or pile yarn (in the case of SPY). The generalized weave design is modified to achieve the desired fabric structure by calculating the number of picks required for each fabric section based on the required geometric parameters. The resulting fabric structure is shown in Figure 11 [104].

**Figure 12:**
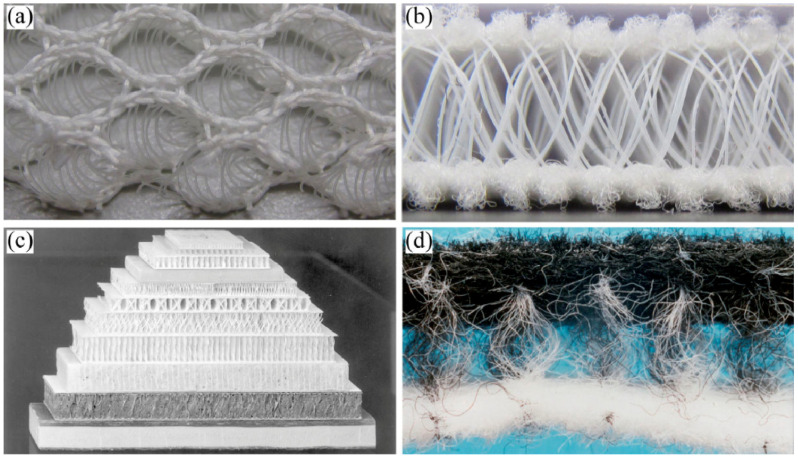
Different types of spacer fabrics: (**a**) warp-knitted; (**b**) weft-knitted; (**c**) woven; (**d**) nonwovens [105].

There have been significant advances in spacer fabrics in recent years, resulting in a range of specialized textile products suitable for different applications. These technical textile innovations have led to the emergence of spacer fabric as a versatile material capable of replacing traditional materials such as polyurethane foam in a variety of applications, including car seats, wheelchairs, sofas, and mattresses. The increasing prominence of technical textiles has driven the adoption of spacer fabrics due to their superior performance and added value. These 3D spacer structures are typically manufactured using braiding, weft-knitting, and warp-knitting techniques, as shown in Figure 12.

**Figure 13:**
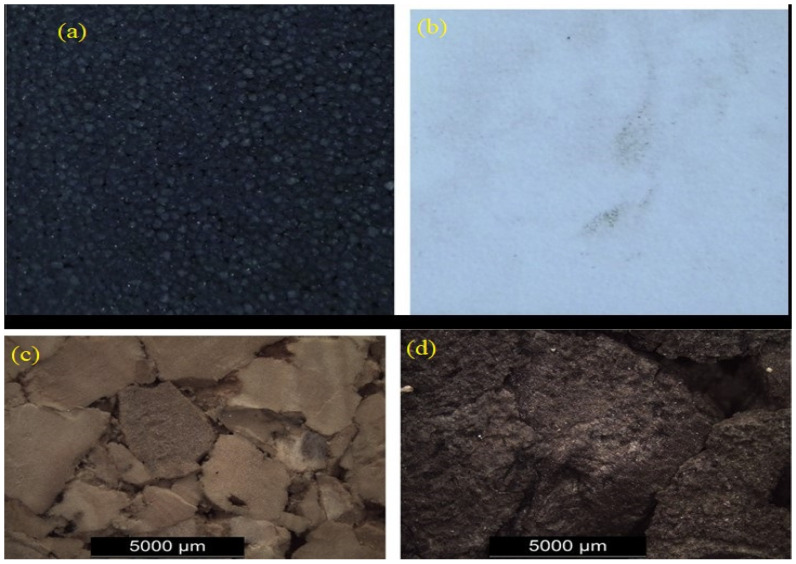
(**a**) Black EPS with a density of 90 kg/m^3^; (**b**) White EPP with a density of 60 and 90 kg/m^3^; (**c**) agglomerated with a density of 199 and 2016 kg/m^3^; and (**d**) Expanded black with a density of 159 kg/m^3^ [106].

Fernandes et al. (2015) [106] state that Peti-bol (EPP and EPS), Sofalca (EC), and CORKSRIBAS (AC), all Portuguese companies, provided samples for the testing of the mechanical properties of synthetic EPP and EPS in comparison to natural cork cellular material, as shown in Figure 13.

**Figure 14:**
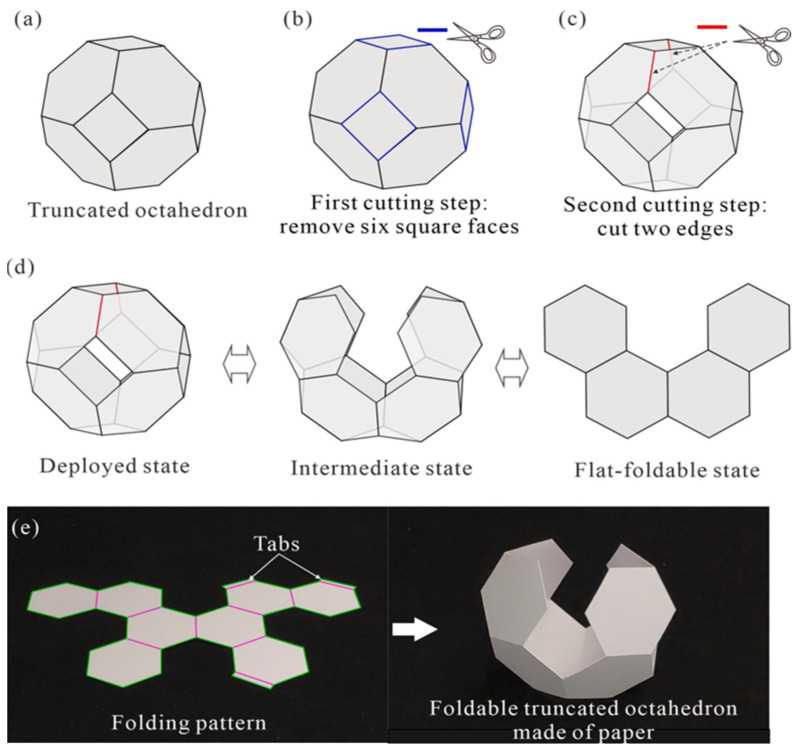
The construction of a foldable truncated octahedron. (**a**) A truncated octahedron. (**b**) First cutting step. (**c**) Second cutting step. (**d**) Folding process of the foldable truncated octahedron. (**e**) Pictures of a foldable truncated octahedron made of 0.3 mm thick card [44].

Creating an Archimedean solid includes the square and hexagonal facets that can be folded into an octahedron [44]. Its edges were sliced to make it fold flat. The square facets are cut away in two stages, and the edges connecting them are trimmed. With only one degree of freedom, the resulting foldable shape rigorously converts from three dimensions to two. During fabrication, a 0.3 mm thick card is used, machine-cut creases and contours are created, and double-sided tape is used for assembly. The construction of the truncated octahedron process is shown in Figure 14 [44].

**Figure 15:**
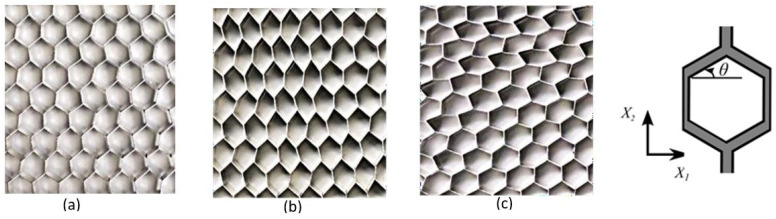
Programmable aluminum base material (**a**) before programming (**b**) after programming in X_1_ direction (**c**) after Programming in X_2_ direction [38].

The very specific case of the cellular solid is a microstructure having a longitudinal direction, where material distribution is constant and the perpendicular cross-section to this direction includes some specific and regular geometrical patterns Figure 15. They are similar to the fiber-reinforced structures in composite materials engineering, but they are formed using a single material. Here, a periodic cellular solid is made up by repeating the unit cells [84,107,108,109], which are the Representative Volume Elements (RVE) frequently discussed in the context of the homogenization method. They can be formed by the so-called programming procedures, and some of the programming imperfections appearing in different research studies are shown in Table 6.

**Figure 16:**
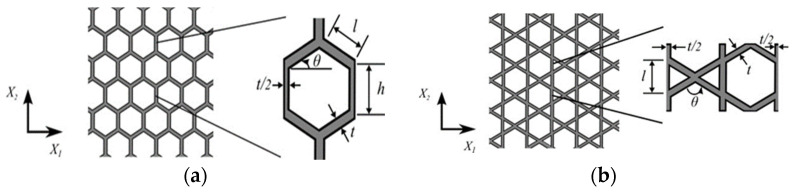
Cellular material honeycombing is (**a**) bending-dominated honeycomb with a hexagonal unit cell and (**b**) stretching-dominated honeycomb with the kagome unit cell [38].

According to the model delivered by Restrepo et al., 2016 [38], two types of programmable materials are a stretching-dominated honeycomb with the kagome unit cell and a bending-dominated honeycomb with a hexagonal unit cell, as shown in Figure 16. The base material used was a shape memory polymer. Shape memory polymers can return to their original shape after being deformed when exposed to a specific stimulus, such as heat [110,111]. This study considers an aluminum honeycomb made from sheets of 5052 alloy that are 0.004 inches. The honeycomb has nominally hexagonal cells that are 1/4 inch in size [38].

**Table 6:** Imperfections on the Cellular materials.

Types of Material	Geometry Features	Imperfection Range	References
Architected Cellular	Overlapping nodes (Edge misalignment, Node displacement)	Variable	[112]
Porous Metals	Void distribution (Pore aspect ratio, Pore alignment)	Variable	[113]
Foams	Cell size variation (Cell shape deviation, Cell wall thickness)	Variable	[114]
Wood-Based Composites	Fiber orientation (Fiber diameter, Fiber length)	Variable	[65]
Carbon Aerogel	Pore size distribution (Pore connectivity, Wall thickness)	Variable	[115]

## 3. Homogenization Methodology

It is widely known that the calculation of the effective (homogenized) material characteristics of cellular solids may proceed in a way similar to that of periodic composite materials. One may use an equity of deformation energy for the homogenized and original solid to determine effective bulk and shear moduli or to directly determine the components of the effective elasticity (material) tensor. This homogenization procedure seems to be even easier for the cellular media due to their programmable distribution of the skeleton made of the same isotropic elastic solid. Two types of programmable material Restrepo et al., 2016 [38], (hexagonal (H) material and Kagome (K) material system) are most frequently considered in the literature. Their effective properties can be defined by some analytical expressions given below, and these are, in turn, effective mass density (ρ), effective elastic modulus in two perpendicular directions (X1, X2), and yield strength.

(1) ρr=ρ*ρs=tlhl+22cos⁡θ(hl+sin⁡θ)

(2) $$\frac{{E_{2}}^{*}}{E_{s}}={\left(\frac{t}{l}\right)}^{3}\frac{\left(h∕I+\sin\theta \right)}{\cos^{3}\theta }\;(X1\;\mathrm{direction})$$

(3) $$\frac{E_{1}^{*}}{E_{s}}={\left(\frac{t}{1}\right)}^{3}\frac{\cos\theta }{h/I\;\sin^{2}\theta }\;(X2\;\mathrm{direction})$$

(4) $$\frac{{\left(\sigma_{p}^{*}\right)}_{2}}{\sigma_{y_{s}}}={\left(\frac{t}{I}\right)}^{2}\frac{1}{2\cos^{2}\theta }$$

where $E_{s}$ is elastic modulus, $\sigma_{y_{s}}$ is yield strength, $\rho_{s}$ is density, t is thickness, l is length of wall and θ is angle between joint shown in Figure 16. Similarly, in the case of the Kagome, $E_{s}$ (Elastic Modulus) and $\rho_{s}$ (density) are expressed as [38]:

(5) $$\rho_{r}=\frac{\rho^{*}}{\rho_{s}}=\frac{2\sqrt{3}}{1-\cos\left(60+\theta \right)}\;\frac{t}{I}$$

(6) $$E^{*}=4\sqrt{3}\;\frac{k_{h}}{I^{2}\left(1+\cos\left(60+\theta \right)\right)}$$

where a specific values of the modulus Kh = 8.5754 N⋅m/rad can be determined by some FEM simulations. An et al. [43] observed that the static property of 3D acoustic metamaterial (AM) based on lattice structures shown in Figure 2 can be analytically defined using the relative density as [43]:

(7) $$\rho^{*}=\frac{3\pi r^{2}+2\sqrt{3\pi r^{2}}+2\sqrt{3\pi \;}\;R^{2}}{a^{2}}$$

Meanwhile, the mechanical property of 3D acoustic metamaterial (AM) was calculated by [116], whereas the strain energy ($k_{\varepsilon }$) with deformation component are applied as [43]:

(8) $$k_{\varepsilon }=\frac{1}{V}B_{\varepsilon }^{T}(D_{a}^{T}k_{uc}D_{a})B_{\varepsilon }$$

(9) $$D_{a}=B_{0}(-\left(B_{0}^{T}k_{uC}B_{0})+B_{0}^{T}k_{uC}B_{a}\right)+B_{a}$$

V (volume of the unit cell) equals $a^{3}$, $k_{uc}$ is the stiffness matrix of this cell, “a” is its edge length, and R is the radius marked in Figure 2. The bulk modulus ($k^{*})$ and buckling strength of such a 3D acoustic metamaterial (AM) can be defined as [43]

(10) $$k^{*}=\alpha +2\beta$$

(11) $$\left(k_{\mathrm{uc}}-\lambda k_{\mathrm{uc}\sigma }\right)d=0$$

where $k_{uc\sigma }$ stands for the stiffness matrix in global coordinates.

Further, Redenbach [46] proposed some statistical approach, where the sphere or volume (v_s_) may follow lognormal and/or gamma probability distributions. This volume having lognormal distribution ($g\left(v_{S}\right)$) equals

(12) $$g\;\left(v_{S}\right)=\frac{1}{\surd 2\pi \sigma v_{S}}\;e^{-\frac{{(\log v_{S}-\mu )}^{2}}{2\sigma^{2}}\;v_{S}\;\geq \;0}$$

or in case of gamma ($f\;\left(v_{S}\right)$) is expressed as

(13) $$f\;\left(v_{S}\right)=\frac{1}{\theta^{k}\Gamma (k)}v_{s}^{k-1}\;e^{-\frac{v_{S}}{\theta }\;v_{S}\;\geq \;0}$$

where K and θ are some shape parameters.

Further, according to the Kirigami-inspired foldable 3D cellular structures, for spherical linkage, the loop closure equation is engaged [44]:

(14) $$Q_{21}Q_{32}Q_{43}Q_{14}=I_{3}$$

The additional kinematic equation for spherical linkage is used according to [44] as

(15) $$\varphi 3=\varphi 5=\varphi 7=\varphi 9=\varphi 11=\varphi 1,\;\varphi 2=\varphi 4=\varphi 6=\varphi 8=\varphi 10=\varphi 12=2\cot^{-1}(\frac{1}{2}\tan\frac{\varphi_{1}}{2})$$

where the applicable Transformation Matrix is proposed as [44]:

(16) $$Q_{\left(i+1\right)}=\left[\begin{array}{ccc} \cos\theta_{i} & -\cos\alpha_{i(i+1)}\sin\theta_{i} & \sin\alpha_{i(i+1)}\\ \sin\theta_{i} & \cos\alpha_{i(i+1)}\sin\theta_{i} & -\sin\alpha_{i(i+1)}\cos\theta_{i}\\ 0 & \sin\alpha_{i(i+1)} & \cos\alpha_{i(i+1)} \end{array}\right]$$

with φ being a dihedral angle.

Close to some analytical models, a variety of the numerical approaches have been delivered in this area and particularly, Nguyen & Noels [35] addressed an issue of the microscopic and macroscopic instabilities in cellular materials, specifically focusing on the microstructure of hexagonal honeycomb structures. This study employs the discontinuous Galerkin method on a macroscopic scale and the finite element method on a microscopic scale. The instability in both cases is resolved using the arc length path and one obtained at the macroscopic-scale

(17) $$\Delta {\bar{U}}_{n+1}=\Delta {\bar{U}}_{n+1}+\delta {\bar{U}}^{\;k}$$

(18) $${\bar{\Delta \mu }}_{n+1}=\Delta {\bar{\mu }}_{n+1}+\delta {\bar{\mu }}^{\;k}$$

where $\Delta {\bar{U}}_{n+1}$ is the macroscopic-scale load correction parameter, ${\bar{\Delta \mu }}_{n+1}$ is the macroscopic-scale displacement correction parameter, $\delta \bar{u}$ stands for the macroscopic-scale correction increment and K is traditionally stiffness matrix. One provides the following statements at the microscopic-scale

(19) $$\Delta {\bar{U}}_{n^{\prime }+1}=\Delta {\bar{U}}_{n^{\prime }+1}+\delta {\bar{U}}^{\;k^{\prime }}$$

(20) $${\bar{\Delta \mu }}_{n^{\prime }+1}=\Delta {\bar{\mu }}_{n^{\prime }+1}+\delta {\bar{\mu }}^{\;k^{\prime }}$$

where $\Delta {\bar{U}}_{n^{\prime }+1}$ is the microscopic-scale load correction parameter, ${\bar{\Delta \mu }}_{n+1}$ is the microscopic-scale displacement correction parameter, $\delta \bar{u}$ is analogous microscopic-scale correction increment and k′ denotes the microscopic stiffness.

Further, some elastoplastic models have been developed (Ling et al. [47]) where a definition of the von Mises yielding stresses of expanded polystyrene (EPE) was necessary in both 3D

(21) σv=12[σ11−σ222+σ22−σ332+σ33−σ112+6σ122+σ232+σ312]

and 2D models

(22) $$\sigma_{v}=\sqrt{\left(\sigma_{c}^{2}+3\sigma_{s}^{2}\right)}$$

where $\sigma_{c}$ is compressive and $\sigma_{v}$ is shear stresses

Zheng et al. [41] proposed another approach, where the conversion of the mass and momentum across the shack front according to continuum-based stress theory as

(23) $$v_{B}\left(t\right)-\;v_{A}\left(t\right)=-\phi (t)(\varepsilon_{B}\left(t\right)-\varepsilon_{B}\left(t\right))$$

and

(24) $$\sigma_{B}\left(t\right)-\sigma_{B}\left(t\right)=-\rho_{0}\phi (t)(v_{B}\left(t\right)-v_{B}\left(t\right))$$

A combination of the above relations (23) with (24) results in

(25) $$\sigma_{B}\left(t\right)=\sigma_{A}\left(t\right)+\frac{\rho_{0}{\left(v_{B}\left(t\right)-v_{A}\left(t\right)\right)}^{2}}{\varepsilon_{B}\left(t\right)-\varepsilon_{A}\left(t\right)}$$

where ϕ(t) is shock front speed, $v_{A},\varepsilon_{A}$ and $\sigma_{A}$ physical quantity ahead of the shock front and $v_{B},\varepsilon_{B}$ and $\sigma_{B}$ are behind the shock front.

Later on, Zargarian et al. [49] introduced the model, where mechanical property of cellular material in term of density can be expressed as

(26) $$\frac{P^{*}}{P_{s}}=C{\left(\frac{\rho^{*}}{\rho_{s}}\right)}^{n}$$

The above Equation (26) can be expressed for Young’s Modulus as

(27) $$\frac{E^{*}}{E_{s}}=c_{1}{\left(\frac{\rho^{*}}{\rho_{s}}\right)}^{n}$$

The analytical derivation for Young’s modulus as a function of relative density of tetra-kai-decahedral unit cell in the following form:

(28) $$\frac{E^{*}}{E_{s}}={\frac{2}{3}c}_{2}{\left(\frac{\rho^{*}}{\rho_{s}}\right)}^{2}{\left(1+C_{2}\left(\frac{\rho^{*}}{\rho_{s}}\right)\right)}^{-1}$$

and also the equation for the Poisson’s ratio as [49]:

(29) $$\vartheta =\frac{1}{2}\;\left(\frac{1-C_{2}\left(\rho^{*}/\rho_{s}\right)}{I+C_{2}\left(\rho^{*}/\rho_{s}\right)}\right)$$

where $P^{*}$ and $P_{s}$ are the properties of cellular and bulk material, similarly and $\rho^{*}\;and\;\rho_{s}$ are densities of cellular and bulk material; C and n are some constants that depend upon the topology of cell and shape of the walls.

Some nonlinear models have been recently developed and reported in [50]. The constitutive equations can be composed with three different contributions, namely

- in linear elastic region:
  (30) $$\sigma =E_{\epsilon }\;\mathrm{if}\;\sigma \leq \sigma_{\mathrm{yield}}$$
- plateau region:
  (31) $$\sigma =\sigma_{\mathrm{yield}}\;\mathrm{if}\;\varepsilon_{\mathrm{yield}}\leq \varepsilon \;\leq \varepsilon_{D\;}(1-D^{-\frac{1}{m}})$$
- and also densification region:
  (32) $$\sigma =\sigma_{\mathrm{yield}}\frac{1}{D}\;{\left(\frac{\varepsilon_{D}}{\varepsilon_{D}-\varepsilon }\right)}^{m}\;\mathrm{if}\;\varepsilon >\varepsilon_{D}(1-D^{-\frac{1}{m}})$$

Further, Rush model [50] assumes power function:

(33) $$\sigma =A\varepsilon^{m}+B\varepsilon^{n}\;\mathrm{with}\;0<m<1,\;1<n<\infty$$

whereas the Gibson proposition [50] is based upon the following representation:

(34) $$\sigma =\sigma_{\mathrm{yield}}+h\varepsilon$$

New empirical model has been also recommended [50]

(35) σ=A1−ⅇ−EAε1−εm+B ε1−εn

and the specific energy W and efficiency E can be expressed analytically as the functions of strain [50]

(36) $$\begin{array}{l} W=\frac{C_{1,A}\rho^{c_{2,A}}}{m+1}\;\varepsilon^{m+1}+\frac{C_{1,B}\rho^{c_{2,B}}}{n+1}\;\varepsilon^{n+1},\\ E=\frac{(\;C_{1,A}\rho^{c_{2,A}}/({m+1))\varepsilon }^{m+1}+(C_{1,B}\rho^{c_{2,B}}/n+1))\;\varepsilon^{n+1}}{C_{1,A}\rho^{c_{2,A}}\varepsilon^{m+1}+C_{1,B}\rho^{c_{2,B}}\varepsilon^{n+1}} \end{array}$$

where σ is the engineering stress, ε is engineering strain, $\sigma_{yield}$ is yield stress, and $\varepsilon_{D}$ is strain value characteristic of the densification phase of D and m. A and B density are dependent parameter while m and n are not.

Moreover, Ohno, Okumura and Noguchi [34] used update Lagrangean formulation, which can be expressed in terms of macroscopic strain rate, macroscopic spin and rigid translation

(37) $${\dot{u}}_{i}^{0}\left(y,t\right)=\left[{\dot{\varepsilon }}_{\mathrm{ij}}^{.\;0}\;\left(t\right)+{\dot{\omega }}_{\mathrm{ij}}^{\;0}\left(t\right)\right]y_{j}+{\dot{c}}_{i}^{.\;0}\left(t\right)$$

where ${\dot{u}}_{i}^{0}\left(y,t\right)$ corresponds to the Lagrangean formulation ${\dot{\varepsilon }}_{ij}^{.0}\left(t\right)$ is macroscopic strain rate, ${\dot{\omega }}_{ij}^{0}\left(t\right)$ is macroscopic spin and ${\dot{c}}_{i}^{.0}(t)$ is translation rate. The principle of virtual work for the unit cell of an infinite periodic material in macroscopically uniform deformation is provided in this approach as

(38) $$\frac{1}{\left|Y\right|}\int_{y}{\dot{\pi }}_{\mathrm{ji}}\delta {\dot{u}}_{{i,}_{j}}\mathrm{dY}=\dot{\Pi_{i,j}}\delta {{\dot{u}}^{0}}_{{i,}_{j}}$$

where the integral $\int_{y}{\dot{\pi }}_{ji}\delta {\dot{u}}_{{i,}_{j}}$ represents the microscopic virtual work n rate as conducted, and $\dot{\Pi_{i,j}}$ is macroscopic work conducted.

According to Chai et al. [39], the poly-methacrylimide (PMI) foam cell morphology was characterized by gyration tensor as follows:

(39) $$G_{\alpha \beta }=\frac{1}{V_{m}}\;\sum_{i=1}^{V_{m}}(r_{\alpha i}^{\left(m\right)}-r_{\alpha }^{\left(b\right)})\;(r_{\beta i}^{\left(m\right)}-r_{\beta i}^{\left(b\right)})$$

where $G_{\alpha \beta }$ is Gaussian function, $r_{\alpha i}^{\left(m\right)}$ and $r_{\alpha }^{\left(b\right)}$ are coordinates of voxel i, m is barycenter of cell and r is gyration tensor. The effective properties on relative density is represented by scaling law:

(40) $$\frac{E^{*}}{E_{s}}=C{\left(\bar{\rho }\right)}^{n}=c{\left(\frac{\rho^{*}}{\rho_{s}}\right)}^{n}$$

where c and n is fitting parameters, $E^{*}$ is property of cellular solid, $E_{s}$ is property of constituent material, $\bar{\rho }$ is relative density and $\rho^{*}$ is density cellular solid.

The very recently Rajakareyar et al., 2023 [37] delivered the asymptotic homogenization process based on the double-scale expansion theory [117,118,119,120,121,122], the homogenized macroscopic elasticity tensor can be expressed as:

(41) $$E_{\mathrm{ijkl}}^{H}=\frac{1}{\left|V\right|}\int vE_{\mathrm{pqrs}}\left(\varepsilon_{\mathrm{pq}}^{0\left(\mathrm{ij}\right)}-\varepsilon_{\mathrm{pq}}^{\left(\mathrm{ij}\right)}\right)\left(\varepsilon_{\mathrm{rs}}^{0\left(\mathrm{kl}\right)}-\varepsilon_{\mathrm{rs}}^{\left(\mathrm{kl}\right)}\right)\mathrm{dV}\;i,j,k,l,p,q,s\;\in \left\{1,2,3\right\}$$

where V stands for the volume of the based RVE, $E_{\mathrm{pqrs}}$ is the locally varying stiffness tensor, $\varepsilon_{\mathrm{pq}}^{0\left(\mathrm{ij}\right)}$ represents the macroscopic strain, and $\varepsilon_{\mathrm{pq}}^{\left(\mathrm{ij}\right)}$ is the microscopic strain (locally periodic). For more detail in 2D and 3D voxels can be expressed as:

(42) $$\varepsilon_{\mathrm{pq}}^{\left(\mathrm{ij}\right)}=\varepsilon_{\mathrm{pq}}\left(x^{\mathrm{ij}}\right)=\frac{1}{2}\;\left(x_{p,q}^{\mathrm{ij}}+x_{q,\;p}^{\mathrm{ji}}\right),\;p,q\;\in \left\{1,2,3\right\}$$

Based on displacement fields $x^{\mathrm{ij}}$, which are found by solving the elasticity equations with the prescribed macroscopic strains:

(43) $${\int v}^{E_{\mathrm{ijpq}\varepsilon_{\mathrm{ij}}}}\left(v\right)\varepsilon_{\mathrm{pq}}(x^{\mathrm{kl}})\mathrm{dV}=\int v^{E_{\mathrm{ijpq}\varepsilon_{\mathrm{ij}}}}(v)\;\varepsilon_{\mathrm{pq}}^{0(\mathrm{kl})}\;\mathrm{dV}\;i,j,l,p,q\;\in \left\{1,2,3\right\}$$

where v is the virtual displacement field.

Theoretically, the homogenized elasticity tensor ($\left[C_{\mathrm{ij}}^{H}\right]$) in the Voigt notation for an orthotropic lattice cell takes the form as [37]:

(44) $$\left[C_{\mathrm{ij}}^{H}\right]=\left[\begin{array}{cccccc} C_{11} & C_{12} & C_{13} & 0 & 0 & 0\\ C_{21} & C_{22} & C_{23} & 0 & 0 & 0\\ C_{31} & C_{32} & C_{33} & 0 & 0 & 0\\ 0 & 0 & 0 & C_{44} & 0 & 0\\ 0 & 0 & 0 & 0 & C_{55} & 0\\ 0 & 0 & 0 & 0 & 0 & C_{66} \end{array}\right]\;i,j\;\in \left\{1,2,3\right\}$$

Let us note by the way that if the given programmed microstructure is too complex for development of analytical formulas relevant to the homogenized isotropic material, then it is possible to engage the Finite Element Method system to make 2D or 3D discretization using solid elements, to apply kinematic periodicity conditions on the outer edges of such a unit cell, and to simulate uni- or bi-directional extension as well as shear deformation of this periodic element. This methodology has been extensively studied in the context of various composite materials and may be applied here with no modifications.

FEniCS is a popular open-source computing platform for solving partial differential equations (PDEs). It can be used to model cellular materials. In the FEniCS, problems involving different materials are handled by defining subdomains within a domain. FEniCS also supports defining complex subdomains and implements variable coefficients [123]. According to Bleyer, 2020, macroscopic stiffness and the homogenization formula for the material unit cell as:

(45) $$\left[C^{\mathrm{hom}}\right]=\left[\begin{array}{cccc} <E_{0e}>-<{{⋋}^{2}/E}_{0e}>+{<{⋋/E}_{0e}>}^{2}/<{1/E}_{0e}> & <{⋋/E}_{0e}>/{<1/E}_{0e}> & <{⋋/E}_{0e}>/{<1/E}_{0e}> & 0\\ <{⋋/E}_{0e}>/{<1/E}_{0e}> & 1/{<1/E}_{0e}> & <⋋> & 0\\ <{⋋/E}_{0e}>/{<1/E}_{0e}> & <⋋> & E_{0e} & 0\\ 0 & 0 & 0 & 2/<1/\mu > \end{array}\right]$$

(46) $$\left[k\right]={\left[\begin{array}{cc} \frac{12}{<\mu >s^{2}} & 0\\ 0 & \frac{12}{<E_{\mathrm{oe}}>s^{2}} \end{array}\right]}_{\left(x,y\right)}\;$$

where $E_{0e}$ is oedometric modulus, and $C^{\mathrm{hom}}$ is macroscopic stiffness.

Topology optimization of cellular material microstructures is investigated utilizing the Harmony Search (HS) algorithm in conjunction with the Bi-directional Evaluation Structure Optimization (BESO) approach. The bulk (K) or shear (G) modulus cellular material optimization problem is defined as [124]:

(47) $$k=\frac{1}{4}\sum_{i,j=1}^{2}D_{\mathrm{ij}}^{H}\;\mathrm{for}\;2D\;\mathrm{cases}$$

(48) $$k=\frac{1}{9}\sum_{i,j=1}^{3}D_{\mathrm{ij}}^{H}\;\mathrm{for}\;3D\;\mathrm{cases}$$

(49) $$G=D_{33}^{H}\;\mathrm{for}\;2D\;\mathrm{cases}$$

(50) $$G=\frac{1}{3}{(D}_{44}^{H}+D_{55}^{H}+D_{66}^{H})\;\mathrm{for}\;3D\;\mathrm{cases}$$

where D^H^ is homogenized elasticity matrix

The elastic modulus, determined by the Gibson–Ashby scaling law, is formulated as a function of the mechanical properties and relative density of the solid structure in Equation (50). According to this theory, the mechanical properties of porous structures depend on whether they exhibit bending- or stretch-dominated mechanical responses [125].

(51) $$E_{\mathrm{lat}}=cE_{\mathrm{sol}}{\left(\frac{\rho_{\mathrm{cell}}}{\rho_{\mathrm{sol}}}\right)}^{n}$$

where $E_{\mathrm{lat}}$ and $E_{\mathrm{sol}}$ are modulus elasticity of lattice structure and bulk material, $\frac{\rho_{\mathrm{cell}}}{\rho_{\mathrm{sol}}}$ is relative density, and n is coefficient.

An isotropic, nonlinear material model called hyperfoam is used to describe elastomeric foams that behave in a hyperplastic manner. Up to 90% compression strain is permitted for elastic deformation, and it is intended for finite-strain applications. The elastic behaviour of the model is determined by a certain strain energy function as [106]:

(52) $$\tilde{U}=\sum_{i=1}^{N}\frac{2\mu_{i}}{\alpha_{i}^{2}}[\lambda_{1}^{\alpha i}+\lambda_{2}^{\alpha i}+\lambda_{3}^{\alpha i}-3+\frac{1}{\beta_{i}}\;\left(J^{-\alpha_{i}\beta_{i}}-1\right)]$$

Here N is Polynomial order, $\lambda_{i}^{\alpha i}$ are principal stretches. J is elastic volume ratio, $\mu_{i}$ is share moduli, $\alpha_{i}$ and $\beta_{i}$ are curve fitting non-integral exponents.

One also notices that having closed-form analytical equations describing effective properties is very attractive while optimizing (programming) cellular solid microstructure, but it is also very important for further uncertainty analysis. Such equations may just be implemented into computer algebra systems with statistical libraries or just probabilistic features. Then, one can introduce some experimentally motivated probability distributions of the cellular solid design parameters and deliver analytical integration if only the probability integrals do exist; otherwise, Monte–Carlo simulation always allows for statistical estimation (cf. Table 2).

## 4. Experimental Works and Some Manufacturing Method

The experimental work was conducted on programmable cellular material like the thermal-mechanical property of epoxy-based SMP NGDE2 (shape-modified polymer (SMP)) using a universal test machine and extensometer as per ASTM D638. The mechanical property is shown in Table 7 [38]. Also, the Mean and Standard deviation of Kagome (K) and Hexagonal (H) cellular material are shown in Table 8 [38]. Also, the mechanical behavior of hexagonal material programmed at various θ is shown in Table 9 and Table 10 in direction X1 and testing in direction X2(P1T2) in direction X2 and testing in direction X2(P2T2) [38]. For reprogramming, the Modulus for in-plane compression for various materials corresponds to the direct programming shown in Table 11. Similarly, for Kagome (k), material programming in direction X2 and testing in direction X1(P2T1) are shown in Table 12, and the reprogramming result is shown in Table 13 [38].

**Figure 17:**
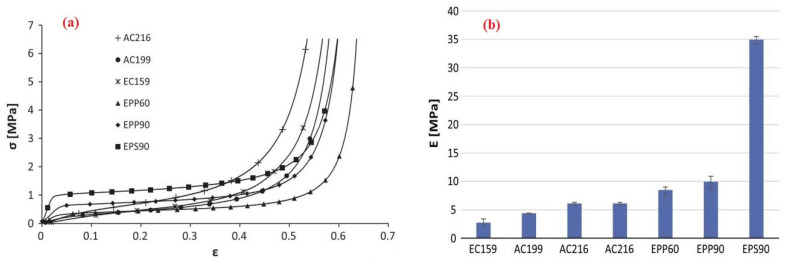
(**a**) Stress–strain curves of synthetic (EPP and ESP) and natural cork (Agglomerated). (**b**) Young Moduli of agglomerated cork and synthetic foams.

The stress–strain graphs demonstrate that synthetic materials (EPP and ESP) exhibit a higher Young’s modulus than agglomerated cork, allowing them to densify at higher strains and reach the plateau zone with less deformation, as shown in Figure 17 [106]. An optimal response for energy absorption consists of a protracted plateau at moderate stress, which is followed by densification at high strain. In comparison to cork, synthetic foams function poorly under repeated impacts. Young’s modulus is lower for cork than for synthetic foams, depending on relative density, as shown in Figure 17 [106].

**Figure 18:**
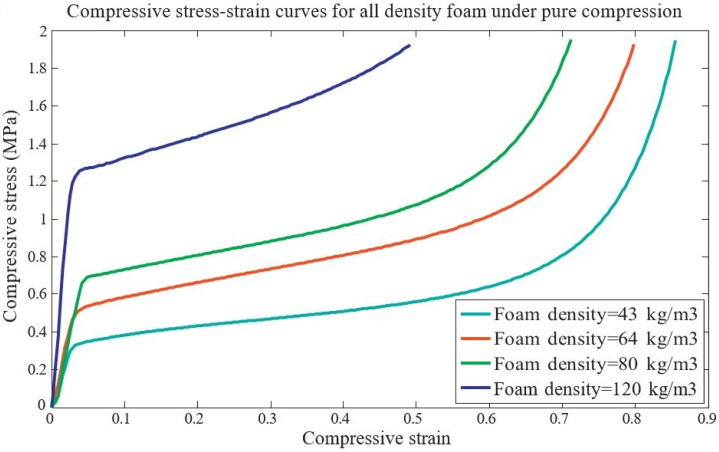
Compressive stress verses strain of EPS foam [48].

**Figure 19:**
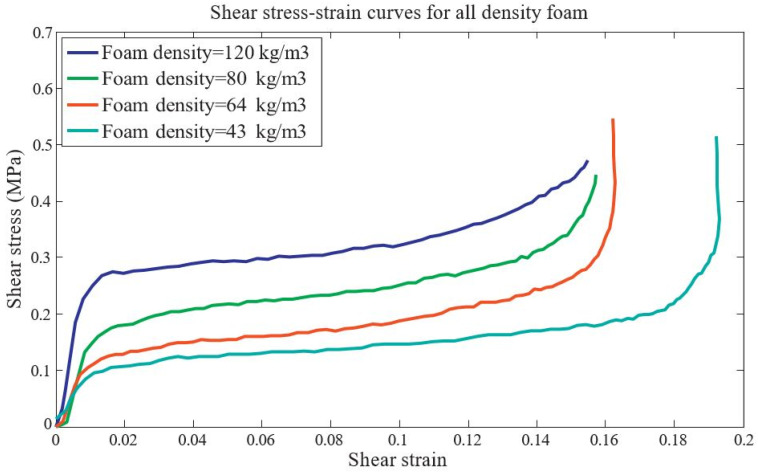
Shear stress verses strain of EPS foam [48].

The mechanical properties of expanded polystyrene (EPS) foam under combined compression and shear loading were examined, as shown in Figure 18 and Figure 19. This study successfully measured EPS foam strain with a DIC strain field measurement system using a specialized device integrated with an INSTRON testing machine. The results show that the yield shear stress is lower compared to the compressive stress, and pure compression tests of four different densities of EPS foams show different compressive stress–strain behavior [48].

**Figure 20:**
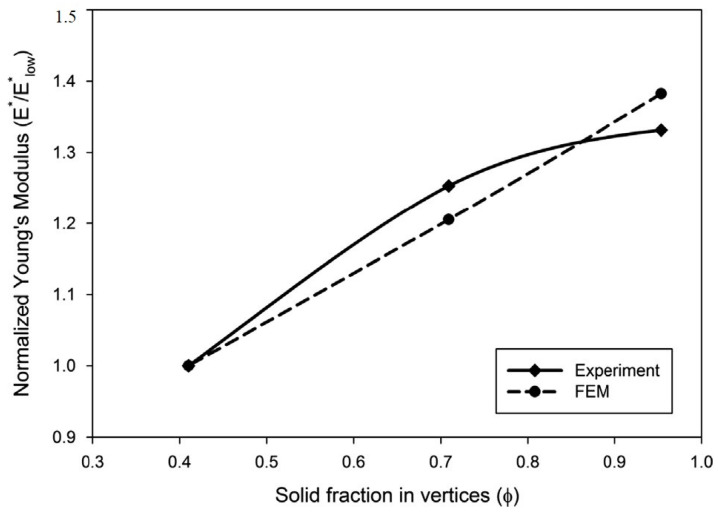
Compression of FEM and Experimental Young’s Modulus [49].

A comparison between finite element method (FEM) predictions of Young’s modulus and experimental measurements is shown in Figure 20, where values are normalized by the modulus of the lowest solid fraction. FEM predicts an increase of 38.2%, while experiments show an increase of 33.1%, which is in good agreement. This experiment was designed to experimentally observe and verify this effect. Although the sample size has an impact on the experimental results, the goal was to validate the numerical simulations and demonstrate that redistributing the material to the vertices significantly increases stiffness. The author recommends expanding the experimental part if facilities permit, to further explore the elastoplastic properties and failure modes, and looks forward to more interesting results [49].

**Figure 21:**
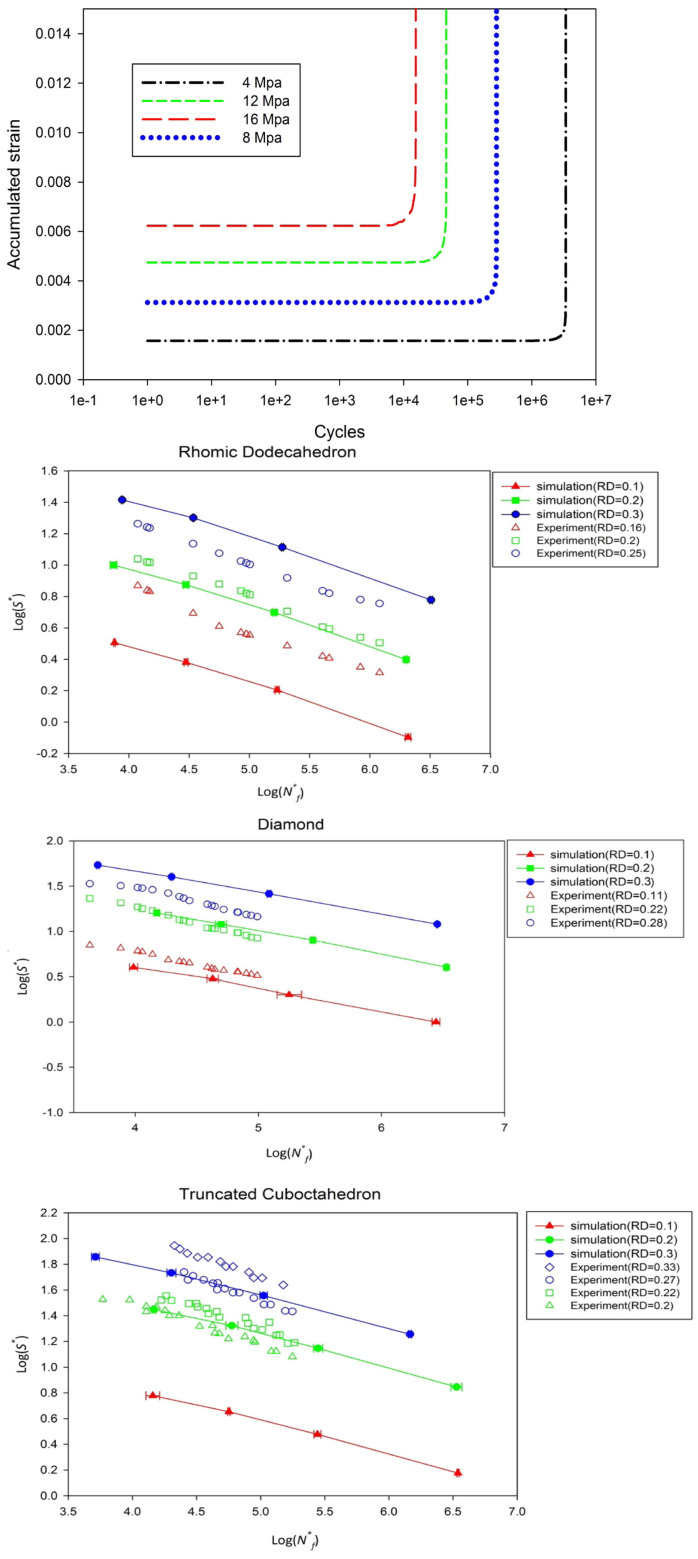
Numerical simulation of the accumulated strain vs. cycles at different alternating stress levels and S-N curves of Titanium scaffolds with different relative densities compared to experimental results for rhombic, diamond, and truncated cuboctahedron structures [126].

The fatigue response of additively manufactured titanium scaffolds with different unit cell geometries and relative densities under cyclic compressive loading was investigated. The failure criterion adopted in the numerical simulations is based on the rapid increase in accumulated macroscopic strain in the representative volume element (RVE), as shown in Figure 21. S-N plots illustrating the fatigue life at different relative densities for each stent geometry were generated (Figure 5). Despite slight differences between simulated and experimental relative densities, there is substantial agreement between the simulated and experimental results. It is worth noting that the predicted fatigue life shortens significantly with increasing stress levels, which is consistent with the experimental results. Both simulated and experimental S-N curves exhibit linear behavior on a logarithmic scale shown in Figure 21, indicating a power–law relationship between uniaxial fatigue strength and fatigue life [126].

**Figure 22:**
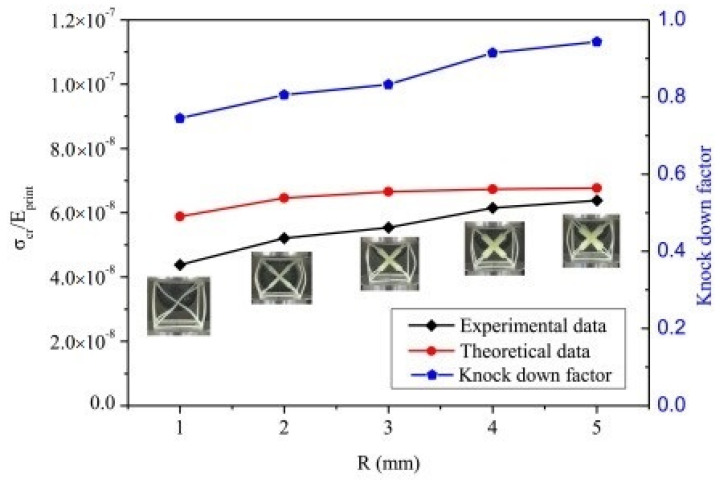
Buckling strength of the 3D AM-based lattice unit-cells with different radii of the resonators [43].

The experimental sample was molded into a solid and fabricated from photosensitive resin with the help of laser-sintering-based 3D printing technology. The compressive test was conducted on a 30 KN Instron (USA) test machine at a 1 mm/minute load for the printed unit cells of 3D acoustic metamaterial-based. The buckling strength change with radius is shown in Figure 22 [43]. The bandgap frequency for different sizes of resonators is calculated by the FE method shown in Table 14 [43].

**Figure 23:**
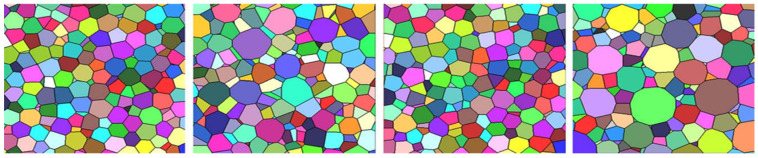
Sections of realizations of the tessellations for the gamma case with parameters VV = 30%, c = 0.2 and 2.0, and VV = 60%, c = 0.2 and 2.0 (from left to right) [46].

**Figure 24:**
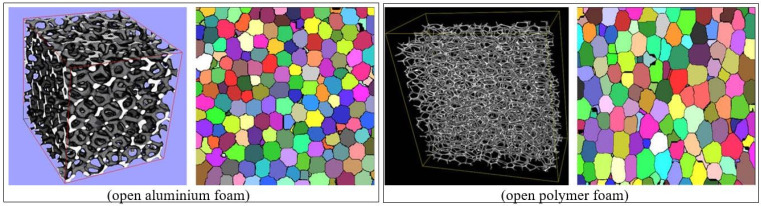
Visualization and sections of foams [46].

All simulation cells were included in the statistics, and a total number of cells equal to 50,000 was taken to investigate each set of parameters, and the tessellation result is shown in Figure 23 [46]. The reconstruction visualization of these two types of foam (open aluminum and open polymer foam) are shown in the Figure 23 and Figure 24.

**Figure 25:**
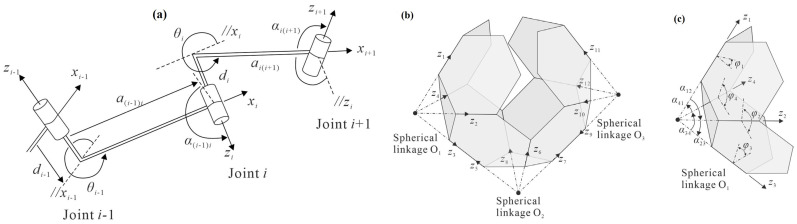
(**a**) A portion of a spatial linkage with coordinate systems under DH notation (**b**) A portion of a spatial linkage with coordinate systems under DH notation, and (**c**) The setup of coordinate systems on the foldable truncated octahedron [44,127].

A specific manufacturing process to automatically manufacture cellular media is the folding process. By using the matrix method of Denavit and Hartenberg (DH) notation shown in Figure 25 of the linkage process of folding [44,127].

**Figure 26:**
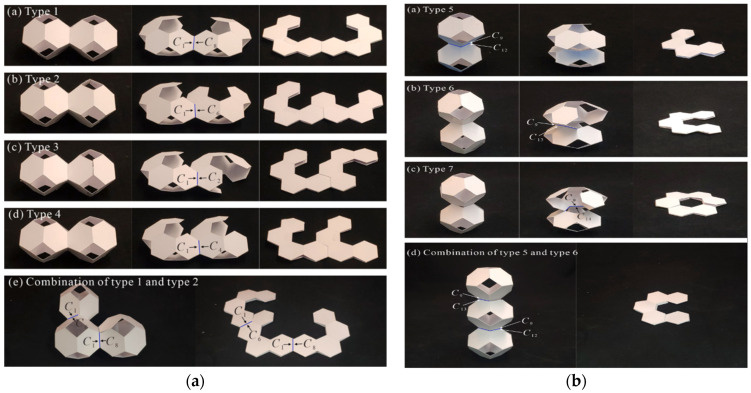
(**a**) Four types of connecting methods for two foldable truncated octahedrons in the horizontal direction (**b**) Three types of connecting methods for two foldable truncated octahedrons in the vertical direction [44].

The physical model of folding processes of seven types is shown in Figure 26 [44].

**Figure 27:**
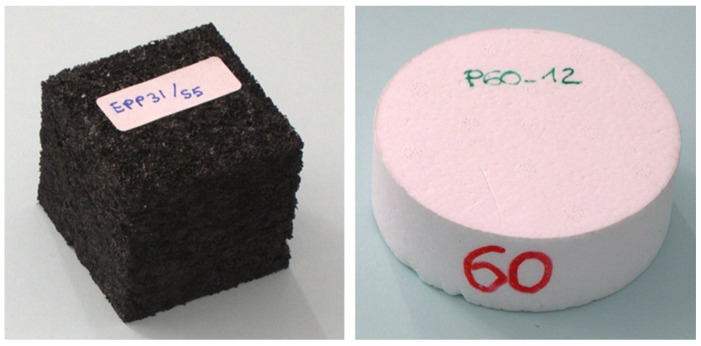
Cubic EPP specimen and cylindrical EPS specimen [50].

Avalle, Belingardi, and Ibba 2007 [50] provided two categories of cellular solids models: phenomenological models and micro-mechanical models. Phenomenological models focus on accurately representing experimental mechanical behavior without directly relating to the physics of the phenomenon. On the other hand, micro-mechanical models analyze the deformation mechanisms of the micro-cell structure under loading. A cubic sample of 50 mm in side length and a cylindrical sample of 100 mm in diameter and 35 mm in height were taken into account for the testing procedure shown in Figure 27.

**Figure 28:**
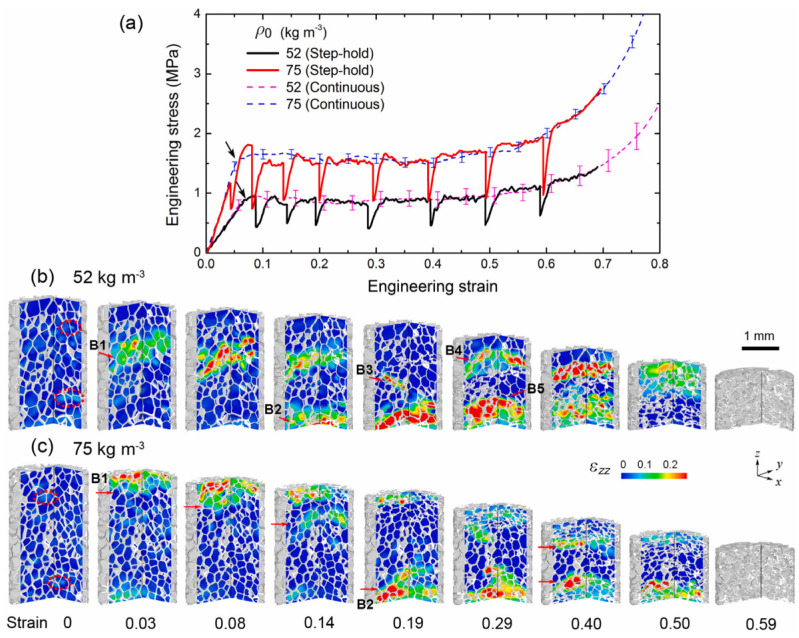
In situ CT testing of the two types of foams (*ρ*_0_ = 52 and 75 kg m^−3^). (**a**) Stress–strain curves. The solid curves with stress drops are from in situ CT test with pauses (step-hold), while the dashed curves are from continuous loading. (**b**,**c**) Volume renderings at different axial strains for the 52 kg m^−3^ and the 75 kg m^−3^ foam samples, respectively. Color-coding refers to axial strain fields obtained via DVC. Positive strain refers to contraction [39].

The stress–strain behavior of the two foams is shown in Figure 28. Positive stress represents compression, and positive strain represents contraction. The solid line with stress drop is derived from in situ CT testing with pauses and coincides with the dashed line with continuous loading. Three stages were identified: pre-collapse, collapse, and densification. Young’s modulus and collapse resistance increase with the increasing initial density of the foam, while the initial densification strain decreases. Collapse strength is defined as the stress at the inflection point between the pre-collapse stage and the collapse stage [39].

## 5. Computer Simulations

**Figure 29:**
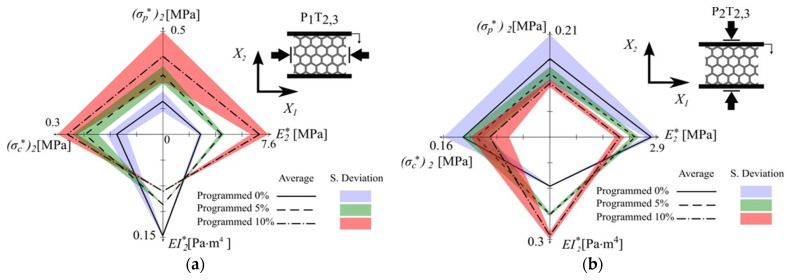
The effective properties for (**a**) material system H under programming and testing modes P1T2 and for (**b**) material system H under programming and testing modes P2T2.

Mechanical properties of programmable cellular material (like Hexagonal (H) and Kagome (K), for instance) may be determined now using a specific numerical simulation [38]. The key problem is the adjacent definition of the Dirichlet and Neumann boundary conditions. In the case of the Hexagonal (H) material system, one may impose such boundary conditions using the directions x1 and x2, as depicted in Figure 29 below [38].

**Figure 30:**
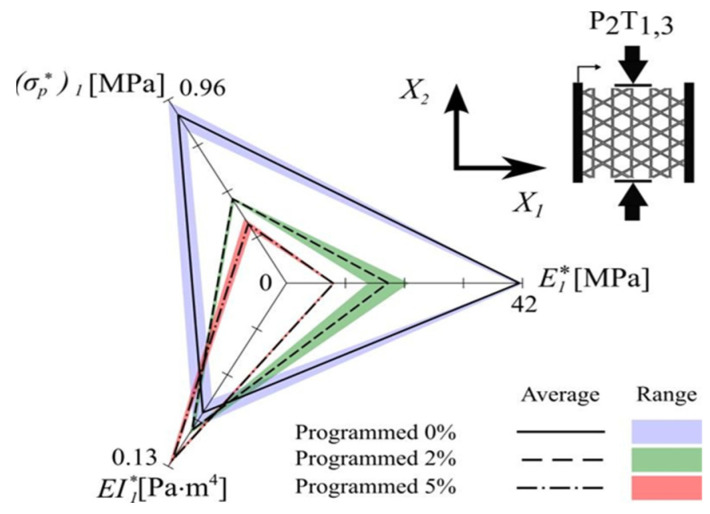
Effective properties for material system K under programming and testing mode P_2_T_1_.

Similarly, in the case of the Kagome (H) material system, numerical analysis was based on the boundary conditions delivered in Figure 30 [38].

**Figure 31:**
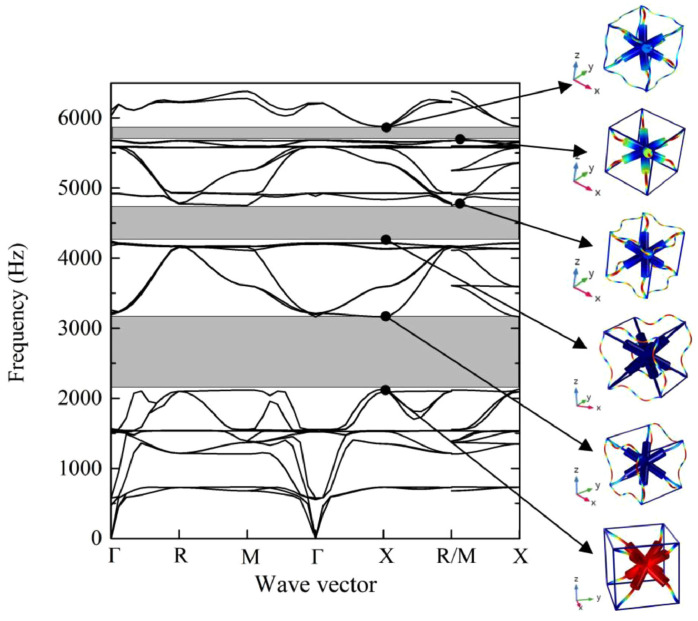
FE simulation for the 3D AM-based lattice structure made of epoxy [43].

**Figure 32:**
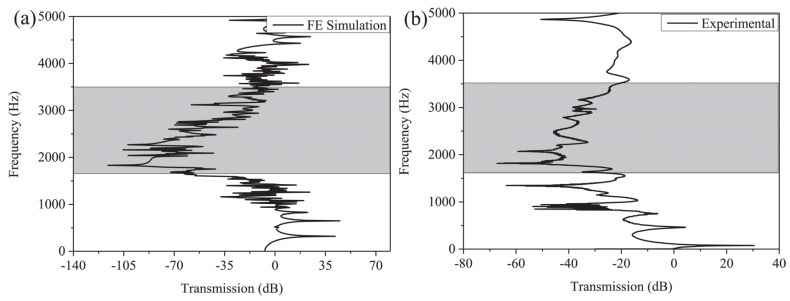
(**a**) FE simulation and (**b**) experimental results for the transmission spectrum of the graded AM-based lattice structure. The gray-shaded regions indicate the frequency ranges where vibration attenuates [43].

The computed band structure of the considered lattice structure is shown in Figure 31, and the mechanical properties of 3D acoustic metamaterial-based materials were also calculated using the FEM approach [116]. This simulation and experimental result of the transmission spectrum for the 3 × 3 × 3-unit cell in 3D acoustic metamaterial-based are shown in Figure 32.

**Figure 33:**
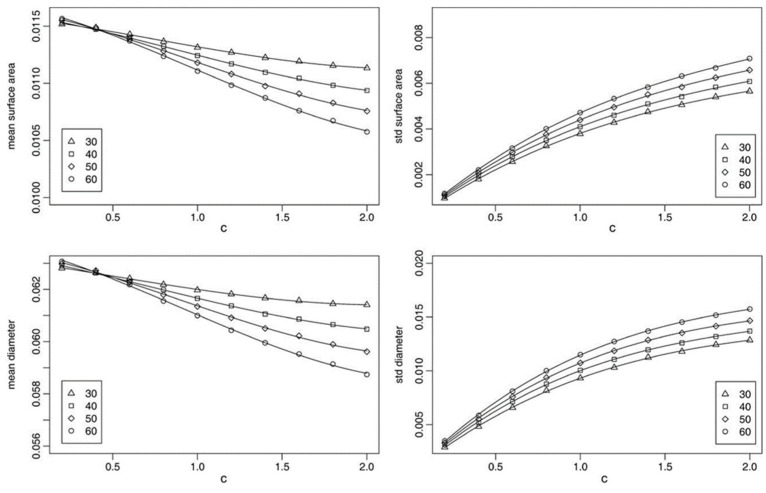
Lognormal case: mean (**left**) and standard deviation (**right**) of the surface area (**top**) and the diameter (**bottom**) of the cells versus c for VV = 30%, 40%, 50%, and 60% (plotted with different symbols) [46].

**Figure 34:**
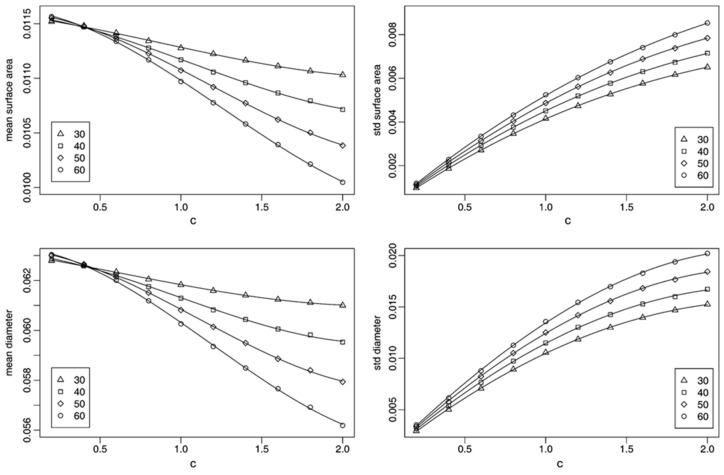
Gamma case: mean (**left**) and standard deviation (**right**) of the surface area (**top**) and the diameter (**bottom**) of the cells versus c for VV = 30%, 40%, 50%, and 60% (plotted with different symbols) [46].

In the simulation procedure of the microstructure of cellular material, random packing of cellular material may also be considered, and this volume may follow lognormal or gamma statistical distributions. The results in both cases are shown in Figure 33 and Figure 34 [46]; where the QHull software 31 August 2020 (8.0.2) was used to compute the results in the form of mean values and standard deviations of the surface area [128].

**Figure 35:**
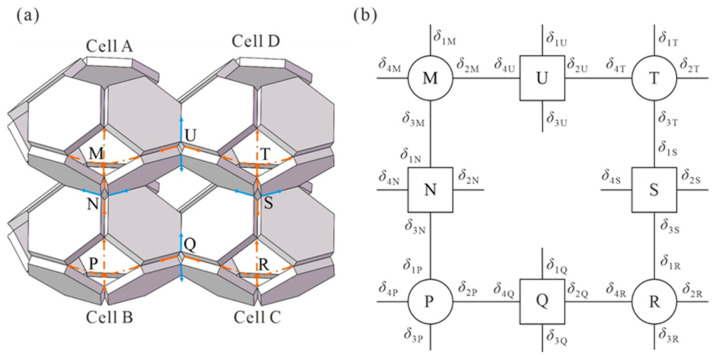
A loop is composed of four thick-panel cells. (**a**) The setup of coordinate systems. (**b**) The schematic diagram of the mobile assembly of spherical 4R linkages and Bennett linkages [44].

The kinematic cell is shown in Figure 35 in a closed loop, and the following transmission loop is presented below [44].

The periodic arrangement of truncated octahedrons in cellular assemblies can be conceptualized as a series of closed-loop mechanisms, each consisting of four interconnected truncated octahedrons. This mechanism is called a kinematic unit and represents the entire cell assembly. By kinematic analysis of this unit alone, one can derive a kinematic model of the entire assembly, specifically focusing on a closed loop consisting of four foldable truncated octahedrons labeled Cell A, Cell B, Cell C, and Cell D. Cells AB and CD are connected by a type 5 connection, while cells BC and DA are connected by a type 4 connection, as shown in Figure 35 [44].

## 6. Uncertainty Analysis Aspects

According to Restrepo et al., 2016 [17], programmed materials’ mechanical properties present uncertainties arising due to several factors during manufacturing and testing. In manufacturing, defects such as broken walls, misalignments, and thickness variations lead to a ±2% uncertainty in the modulus of elasticity due to broken walls. Sample size effects can result in uncertainties ranging from ±1% to ±5% ineffective properties, particularly when sample dimensions are smaller than the critical buckling mode wavelengths. Changes in material symmetry resulting from programming can introduce ±3% uncertainty in certain mechanical properties. The controlled introduction of morphological imperfections during programming can generate up to ±4% uncertainties in the effective modules. Although analytical models have their well-known limitations in predicting the behavior of manufactured samples, finite element simulations can alleviate uncertainties by up to ±2%. Furthermore, exogenous factors, such as friction boundary conditions and strain rate sensitivity, can cause variations of up to ±3% ineffective mechanical properties.

The efficiency and dependability of the suggested periodic truss–lattice constructions were based on the acoustic metamaterial local resonance process [43]. Although whole bandgaps were produced in the three-dimensional lattice structures, the precise mechanisms responsible for the bandgap production remain unclear. Though the exact correlations between these factors and bandgap width were unclear, parametric studies suggest that modifications in some geometric parameters can enlarge the bandgaps [43]. While the impacts of unit-cell size and material properties on the bandgaps were studied, their precise consequences are still unknown [43]. The suggestion of using composite lattice structures to achieve bigger bandgaps raises more questions about their efficacy and applicability. At the same time, there was agreement between simulation findings and experimental validation of the proposed designs; possible limits or discrepancies still needed to be completely addressed. Overall, while the work’s results provide new insights into reducing elastic waves and vibrations, questions about the mechanisms regulating bandgap generation and the viability of suggested designs still need to be answered [43]. More investigation and testing are required to resolve these uncertainties and guarantee the practical effectiveness of the proposed lattice architectures.

According to Redenbach, 2009 [46], the variations seen in Laguerre tessellations are produced by spherically packed spheres with different volume distributions [46]. While tessellations from gamma and lognormal distributions differ little when sphere volumes vary slightly, these differences become more noticeable as sphere volumes vary significantly. Moreover, disparities in geometric properties are shown when Laguerre tessellations are fitted to actual foam structures; these differences may result from production-related restrictions in the foam structure [46]. Examining these restrictions may help us better understand and provide better suggestions for choosing pertinent traits. Additionally, models of foam structures that are helpful for mimicking macroscopic material properties can be created by dilating the tessellations to meet the appropriate volume proportion. The modeling method is made more complex and unpredictable by its capacity to reproduce local variations in structure thickness using locally adaptable dilations.

It focuses on investigating foldable arrays made of truncated octahedrons with facets that have either finite or zero thickness [44]. The arrays were kinematically identical despite differing mechanisms, e.g., Bennett 4R linkages or spherical 4R connections, guaranteeing continuous correlations among dihedral angles along creases. Although the work concentrates on particular connection types, there are possible applications in many other scientific and engineering domains, such as robotic arms and deployable habitats. While this study focused on truncated octahedrons [44], the idea applies to other polyhedrons as well, such as octahedrons and cuboctahedrons. During deployment, this extension presents issues with physical interference and connection conditions. Examining the application of alternative polyhedrons offers a fascinating direction for further theoretical and computational studies.

The uncertainty of the cellular material/solid with modern computation techniques is shown in Table 15.

## 7. Discussion

A three-dimensional truss lattice structure is customized for effective absorption of low-frequency and broadband elastic waves. With the incorporation of local resonance mechanisms, these structures introduce band gaps that effectively hinder elastic wave transmission, which is facilitated by radius-hopping discontinuities in the cross braces of each unit. This innovative approach has practical advantages over traditional acoustic metamaterials. Studying the band structure of these lattice structures can provide insights into the influence of unit cell composition on band gap formation while also proposing composite structures aimed at enlarging the band gap width. In addition, the discussion addresses the enhancement of mechanical properties through targeted adjustments of cross-brace dimensions within the unit. Experimental verification highlights the effectiveness of this design strategy, paving the way for the development of grid truss structures capable of meeting bearing and vibration damping requirements.

Laguerre tessellation is derived from random sphere packing as a potential model for the microstructure of porous or polycrystalline materials, focusing on hard sphere packing with lognormal or gamma-distributed volumes. The geometric characteristics of Laguerre cells vary with the volume fraction of sphere packing and the coefficient of variation of the volume distribution. Polynomial descriptions of certain element characteristic moments are provided, allowing tessellation models to fit real materials without the need for additional simulations.

Inspired by origami and kirigami, create 3D cellular components with rigid faces that can be folded flat using a single degree of freedom. By identifying seven connection types between foldable truncated octahedrons, various 3D honeycomb arrays were constructed, characterized by spherical 4R links or a hybrid of spherical and Bennett 4R links. A thorough kinematic analysis confirmed the single degree of freedom of the array and the kinematic equivalence of thin and thick surfaces. Physical models validated the designs, highlighting their potential applications in deployable space habitats and robotic arms. This innovative approach extends the application of traditional folding technology to advanced, practical structures.

Cellular materials are homogenized using complex multiscale computational homogenization methods focusing on microbuckling and positioning zones during macroscopic loading, using advanced methods including the discontinuous Galerkin method and classical finite element resolution. The aim of the review is to enhance the understanding of cellular material responses and provide valuable insights for structural design optimization.

The dynamic compression and shear tests on EPS foams of varying densities focused on finite strain rates and loading angles due to experimental limitations. In the future it should explore higher strain rates and wider loading angles to better understand foam deformation behavior. Addressing these limitations and expanding the scope of this study could enhance understanding of the mechanical properties of EPS foams for different applications.

The dynamic impact behavior of metal foams, particularly focusing on their shock-like response at high loading rates, It addresses limitations in existing experimental methods and proposes a virtual test method to explore rate sensitivity in cellular materials. By analyzing stress–strain states and deformation modes, this study reveals distinct behavior in cellular materials compared to dense metals. It identifies a unique curve representing dynamic stress–strain states and improves experimental techniques for characterizing rate sensitivity in real-world applications.

Finite element analysis (FEA) has been applied to study the elastic properties of cellular solids. Similarly, in situ X-ray computed tomography was used to explore the 3D microstructure of PMI foams under compression. The cellular structures were studied to highlight their piezoelectric properties. It focuses on periodic cellular lattice structures based on Bravais lattice systems, providing insights into representative volume elements (RVEs) and their mechanical behavior.

The discussions include the development of fabric structures for various applications, advances in spacer fabrics, and modeling of programmable materials such as hexagonal and unit cells. The mechanical properties of these materials were determined through numerical simulations and experimental verification. Restrepo et al. (2016) [38] studied the uncertainty in the mechanical properties of programmed materials due to manufacturing defects and changes in material symmetry. It highlights the importance of finite element simulations in reducing these uncertainties. This review also deals with the fatigue response of additively manufactured titanium stents and the mechanical properties of expanded polystyrene (EPS) foam under different loading conditions. The agreement between finite element predictions and experimental measurements emphasizes the validity of the numerical models used in these studies.

Overall, this review synthesizes theoretical models, numerical simulations, and experimental data to provide a comprehensive understanding of the mechanical properties of cellular materials and aims to guide future research and applications in this field.

## 8. Concluding Remarks

In conclusion, this review highlights the innovative potential of programmable cellular solids in engineering materials, providing tunable mechanical properties through strategic defects in the unit cell. The exploration of periodic lattice structures designed using acoustic metamaterial principles highlights their suitability for vibration control, as well as their lightweight and strong properties. Through band structure analysis, the influence of unit cell composition on band gap formation, as well as the influence of structural and material parameters, are clarified. Stochastic analysis within homogenization holds the promise of improving lattice structure performance by resolving uncertainties associated with manufacturing and environmental factors. Additionally, the research delves into 3D foldable cellular components inspired by origami and kirigami, demonstrating their diverse applications in areas such as space habitats and robotics.

This study provides a comprehensive overview of Laguerre tessellation, demonstrating their potential as microstructural models of cells or polycrystalline materials. These tessellations can provide insights into material geometry by employing random sphere packings with lognormal or gamma-distributed volumes. Polynomial descriptions can be fitted to real materials such as open polymers and aluminum foam, highlighting changes in geometric properties under different filling parameters. Stochastic analysis helps evaluate the variability of these properties, which is critical to understanding factors such as sphere volume distribution and packing density. Furthermore, the homogenization technique establishes a link between microscopic geometry and macroscopic behavior, providing avenues for future research in mechanical property analysis. Stochastic homogenization helps predict the statistical distribution of mechanical parameters, aiding design optimization and reliability assessment. Integration of stochastic analysis and homogenization promises to enhance foam structural design, resulting in improved performance, robustness, and reliability.

We conclude that the second-order computational homogenization framework for cellular materials addresses microbuckling and macropositioning problems by integrating the Mindlin strain gradient continuum at the macroscale and utilizing the discontinuous Galerkin method. It goes beyond classical multiscale schemes and shows effectiveness in capturing microbuckling and localization bands, as demonstrated by uniaxial compression testing of hexagonal honeycomb specimens. Despite the limited drop height and mass, the deformation characteristics of EPS foam can be effectively analyzed using the novel drop weight tower system, providing valuable insights into its mechanical performance under complex loading conditions. Integration of the INSTRON testing machine with the DIC strain field system shows that shear deformation significantly reduces the compressive strength of EPS foam under combined compression and shear loading. Furthermore, a 3D Voronoi-based finite element model successfully studied the dynamic behavior of metal foams under impact, demonstrating its efficacy in capturing the impact-induced mechanical response. Finally, finite element simulations highlighted the influence of solid distribution on the elastic properties of open-cell porous materials. Adjustments in solid distribution affect the elastic modulus and Poisson’s ratio, especially at different relative densities. Experimental validation supports these simulation results, emphasizing the importance of solid distribution in optimizing the mechanical properties of porous materials.

With the future integration of stochastic analysis into homogenization [134,135], there was potential to improve understanding and prediction of how morphological imperfections embedded in periodic cellular solids influence their effective mechanical characteristics. This stochastic approach offers insights into the variability and uncertainty associated with programmable materials, promoting more robust design and manufacturing methodologies. Furthermore, homogenization techniques allow the examination of the macroscopic behavior of these materials, covering imperfections and their influence on mechanical properties, thus advancing the understanding of customized applications.

## Figures and Tables

**Table 1:** Research articles on the prescription of cellular materials.

Cellular Material	Structure	Mechanical Parameter	Performance	Reference
shape memory polymers and shape memory alloys	periodic cellular solids	tensile and creep	mechanical properties, modification, reversibility, repeatability and control of imperfection	[38]
artificial acoustic metamaterial	(3D) truss-lattice structures	bulk modulus, buckling strength, and stiffness-to-mass ratio	bulk modulus and buckling strength increase with radius, while stiffness decreases	[43]
3D Archimedean solid flat using paper folding	origami and kirigami principles	geometrical parameters of spherical and Bennett linkages, dihedral angles	potential engineering applications of foldable cellular structures, such as space habitats and robot arms	[44]
polycrystalline materials	lognormal or gamma distributed volumes	volume, surface area, mean width and number of facets	difference between lognormal or gamma distributed tessellations	[46]
-	hexagonal structure	strain localization and micro-buckling	microstructural instabilities and localization bands	[35]
polystyrene (EPS) foam	-	compressive and shear stresses	foam density and strain-rate	[47,48]
metal foams	3D Voronoi structure	strain, stress ahead of the shock front, and dynamic stress–strain relationship	loading-rate sensitivity mechan, sm of cellular materials	[41]
photopolymer Objet Vero Blue Full Cure M840 material	open-cell structure	Young’s modulus and Poisson’s ratio	good agreement between numerical simulations and experimental results	[49]
various types of foams (EPP, PUR (Bayfill EA), EPS, and PPO/PS (Noryl GTX)	structural foams	Elastic property	introduces new empirical formulations and identifies density-dependent laws	[50]
-	hexagonal honeycomb structure	buckling behavior	establishes a homogenization theory and derived conditions for microscopic symmetric bifurcation	[34]
Polymethacrylimide (PMI) foam	3D microstructures	strength–density, stress–strain curves, buckling strength index of cell walls, and deformation banding	deeper understanding of the mechanical behavior	[39]
Piezoelectrically active cellular solids	hexagonal, tetragonal, and triangular structure	elastic, dielectric, and piezoelectric properties	relationship between cellular structure, deformation modes, and electromechanical properties	[36]

**Table 2:** Modern approach status of stochastic approaches in cellular material.

Stochastic or Probability Method	Cellular Material	Improvement on Mechanical Property	Reference
Monte Carlo Simulation	Aluminum Foam	Compressive Strength	[57]
Monte Carlo Simulation (MCS)-based Stochastic Finite Element Analysis	Wood	Compression Strength	[58]
Hybrid Multilevel Approach based on Excitable Cellular Automata	Ceramic Coating—Polycrystalline Metallic Substrate	Deformation Processes	[42]
Lognormal Random Factor Accounting for Statistical Scatted	Aluminum Alloys (specifically 2024-T351)	Fatigue Crack Growth Rate	[59]
Monte Carlo Simulation (Poisson process)	Foam A	Compressive Strength	[60]
Finite Element Method with Random Fields	porous aluminum	tensile modulus and yield strength	[61]
Artificial Neural Networks for porosity prediction	wood fiber	elastic moduli	[62]
Utilized for modeling nano porous cellular materials using probability density functions	open-porous cellular materials	compressive response	[63]
Stochastic lattice creation techniques	cellular materials properties	heat exchangers and mechanical components	[64]
Stochastic tessellation via Aboav-Weaire law	classification and selection of cellular materials	biomimetic approach	[65]
Stochastic modeling based on μCT-image analysis	open-cell foam	microstructure complexity and mechanical performance	[66]
Stochastic models	cellular materials imperfection (ti6al4v cellular structures)	mechanical property of 2d structure (elastic modulus and strength)	[67]
Finite element models	metal foams	dynamic stress–strain relationships and exploring energy absorption	[41]
lognormal distribution	Polymethacrylimide (PMI) foam	analytical constitutive model	[39]

**Table 3:** Difference between open and closed cell foam.

Open Cell Foam	Close Cell Foam	Numerical Modeling	Difference in Mechanical Property	Reference
Interconnected porous structure	Closed gas-filled pores	Voronoi tessellation is used to model random polyhedral cells mimicking open-cell foam microstructure	Lower stiffness and strength compared to closed-cell foams	[68,69]
Open-cell foam has open, interconnected cells that absorb water and other substances. It is softer, lighter, and more flexible	Closed-cell foam has closed cells, which seal out water, air, and more. It is denser, more rigid, and more robust	Numerical modeling of open-cell metal foam with a Kelvin cell. Numerical and experimental study of open-cell foams for the characterization of heat exchangers	Open-cell foam is less dense and offers better sound absorption. Closed-cell foam is denser and offers higher strength and insulation.	[70,71,72,73,74]

**Table 4:** Processing techniques for the Microstructure formation of Cellular Material.

Types of Cellular Material	Microstructure Formation Method	Reference
architected cellular materials	additive manufacturing	[85]
hard sphere packings with lognormal or gamma distributions	investigating geometric characteristics	[45]
cellular ceramic composites	3d microarchitecture	[3]
-	cellular automata modeling for deformation processes	[86]
metals and alloys in laser additive manufacturing	laser additive manufacturing	[87]
porous metals	solid freeform fabrication techniques	[88]
-	neural cellular automata	[89]
hexagonal honeycomb cellular material	polynomial interpolation method	[35]
metal foams	3D Voronoi structure	[41]
Poly-methacrylimide (PMI) foam	in situ x-ray micro-computed tomography (CT)	[39]

**Table 5:** Computational techniques for the Microstructure formation of Cellular Material.

Types of Cellular Material	Microstructure Formation Method	Reference
Bio-Inspired Cellular Materials	Artificial Intelligence, Machine Learning, Deep Learning	[93]
Square Core Cellular Materials	Computational Homogenization	[94]
Cellular Metals	X-ray Computed Tomography, Infrared Thermography	[95]
High Entropy Alloys	Ab initio approaches	[96]
Closed-cell cellular materials	Finite shell elements modeling	[97]
Periodic cellular solids	Structural modeling	[98]
3D printed cellular structures	Modeling and characterization	[99]
hexagonal honeycomb cellular material	arc-length path	[35]
metal foams	3D Voronoi technique and discrete deformation gradient method	[41]
3D open-cell structure	3D CAD models	[49]
Poly-methacrylimide (PMI) foam	Digital volume correlation (DVC)	[39]

**Table 7:** Tensile properties of base epoxy SMP. Type I tensile test specimen per ASTM D638. Displacement rates are 0.4 mm/min and 0.05 mm/min. Mean ± standard deviation [38].

Displacement Rate [mm/min]	Es (ε = 0) [MPa]	σ_v_ [MPa]	σ_f_ [MPa]	ε_f_
0.4	1169.8 ± 158.7	27.4 ± 0.4	18.5 ± 0.6	0.3 ± 0.1
0.05	883.1 ± 166.4	19.9 ± 0.7	16.0 ± 0.5	0.4 ± 0.1

**Table 8:** Measured dimensions of the as-fabricated specimen. Mean ± Standard deviation [38].

Material System	l [mm]	h [mm]	t [mm]	b [mm]	θ [o]
H	10.0 ± 0.1	10.0 ± 0.1	1.08 ± 0.3	10.0 ± 0.2	29.4 ± 1.7
K	10.0 ± 0.2	-	0.72 ± 0.1	10.0 ± 0.4	118.7 ± 3.7

**Table 9:** Test summary for the H material system in mode P_1_T_2_. Mean ± standard deviation. The sub-index 2 indicates the testing direction [38].

	θ	E_2_* [MPa]	(σ_p_)_1_* [MPa]	(σ_p_)_1_* [MPa]	El_2_* [Pa.m^4^]
Programmed 0%	29.4 ± 1.7	2.7 ± 0.2	0.16 ± 0.05	0.13 ± 0.03	0.14 ± 1.98 × 10^−3^
Programmed 5%	35.1 ± 3.7	4.2 ± 0.1	0.29 ± 0.04	0.22 ± 0.04	9.97 × 10^−2^ ± 2.45 × 10^−4^
Programmed 10%	38.3 ± 6.1	6.9 ± 0.6	0.38 ± 0.12	0.27 ± 0.03	8.08 × 10^−2^ ± 1.35 × 10^−4^

**Table 10:** Test summary for system H under P_2_T_2_. Mean ± standard deviation [38].

	θ	E_1_* [MPa]	(σ_p_)_2_* [MPa]	(σ_p_)_2_* [MPa]	El_2_* [Pa.m^4^]
Programmed 0%	29.4 ± 1.8	2.7 ± 0.15	0.16 ± 0.05	0.13 ± 0.03	0.14 ± 1.98 × 10^−3^
Programmed 5%	27.1 ± 2.7	2.3 ± 0.11	0.13 ± 0.01	0.11 ± 0.02	0.23 ± 0.01
Programmed 10%	21.4 ± 7.4	1.9 ± 0.13	0.11 ± 0.01	0.09 ± 0.03	0.29 ± 0.01

**Table 11:** Modulus for in-plane compression (E2*) from the re-programming trials on a specimen from the H material system [38].

Programming Cycle	E_2_* [MPa]	E_2_* [MPa] Direct Programing Cycle
0	2.7	2.7 ± 0.5
1	4.1	4.2 ± 0.8
2	2.6	2.7 ± 0.5
3	6.4	6.9 ± 0.3
4	2.5	2.7 ± 0.5

**Table 12:** Test summary for system K under P_2_T_1_. Mean ± standard deviation. The sub-index 1 indicates the testing direction [38].

	θ	E_2_* [MPa]	(σ_p_)_2_* [MPa]	(σ_p_)_2_* [MPa]	El_2_* [Pa.m^4^]
Programmed 0%	118.7 ± 3.8	41.1 ± 0.45	0.88 ± 0.06	−−	9.33 × 10^−2^ ± 7.6 × 10^−3^
Programmed 2%	107.5 ± 6.9	17.9 ± 0.03	0.44 ± 0.01	0.26 ± 0.06	0.10 ± 2.5 × 10^−3^
Programmed 5%	92.3 ± 10.2	8.3 ± 0.18	0.31 ± 0.02	0.28 ± 0.02	0.13 ± 3.7 × 10^−3^

**Table 13:** Modulus for in-plane compression (E_1_*) moduli from the reprogramming trials on a specimen from the K material system [38].

Programming Cycle	E_1_* [MPa]	E_1_* [MPa] Direct Programing Cycle
0	41.1	41.1 ± 0.45
1	17.6	17.8 ± 0.03
2	39.1	41.1 ± 0.45
3	7.9	8.3 ± 0.18
4	36.4	41.1 ± 0.45

**Table 14:** The bandgap frequency ranges calculated by the finite element method for different sizes of resonators [43].

Unit Cell L (cm)	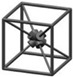	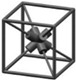	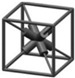	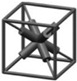	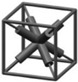	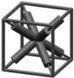
Bandgap (Hz)	2648–3154	1860–2291 3254–3504	1723–2608	1719–2800	4572–4864	1723–2020 2224–2317 8–2580

**Table 15:** Computer simulation verses uncertainty threshold of cellular material/solid.

Computer Simulation Technique	Uncertainty Threshold	Reference
Cellular automata simulations of microstructure evolution	Uncertainty quantification in multiscale material modeling	[129]
Finite Element Analysis (FEA)	Investigates the impact of functionally graded porosity on the energy absorption of metal lattices.	[130]
Coupled FEA and Discrete Element Method (DEM)	Develops a multiscale modeling framework to predict the mechanical response of polymer cellular solids under impact.	[40]
Thresholding segmentation	Thresholding can be used to reduce segmentation errors and uncertainty in material imaging, especially when high contrast is present. Proper selection of threshold values is important. Heterogeneous thresholding may be needed for cellular materials with complex microstructures.	[131]
A multiscale optimization framework considering microscale material uncertainties	Designs optimized assuming spatially varying material uncertainties are up to 74% more robust (in terms of standard deviation of compliance) compared to designs optimized with uniform uncertainties when subjected to spatially varying uncertainties.	[132]
Ensemble methods for molecular dynamics simulations	Ensemble methods play a key role in uncertainty quantification for molecular dynamics simulations of materials. Using 25 replicas with 4 ns of simulation each can often achieve accurate and reproducible results.	[133]
Probabilistic cellular automata simulations, Metamodeling	Quantitative assessment of uncertainties is essential. Advanced statistical methodology is needed for uncertainty propagation and sensitivity analysis.	[134]
Generative Adversarial Networks (GANs)	Utilizes machine learning to optimize the design of cellular materials for bone implant applications	[135]
Finite Difference Time Domain (FDTD) method	Investigates bioinspired cellular designs for manipulating sound waves	[136]
